# Network Pharmacology-Based Exploration of Complementary Molecular Mechanisms of Heat-Clearing (Scutellariae Radix, Coptidis Rhizoma) and Blood-Tonifying Herbs (Angelicae Sinensis Radix, Paeoniae Radix Alba) in Cold Hypersensitivity in Hands and Feet

**DOI:** 10.3390/life16091406

**Published:** 2026-08-25

**Authors:** Eunsu Lee, Yunseo Kim, Jihyun Sang, Hongjae Kim, Jina Youth, Hyeon Seo Kim, Young-Cheol Lee

**Affiliations:** 1 Department of Korean Medicine, College of Korean Medicine, Sangji University, Wonju-si 26339, Republic of Korea; leeeunsue12@gmail.com (E.L.); o3ofall0321@gmail.com (Y.K.); jihyun021102@gmail.com (J.S.); hongjaekim98@gmail.com (H.K.); jinayouth216@gmail.com (J.Y.); 2 Department of Korean Language & Literature, College of Humanities, Sogang University, Seoul 04107, Republic of Korea; hyeonseokim.e@gmail.com; 3 Department of Herbology, College of Korean Medicine, Sangji University, Wonju-si 26339, Republic of Korea

**Keywords:** cold hypersensitivity in hands and feet, heat-clearing herbs, blood-tonifying herbs, network pharmacology, protein–protein interaction, pathway enrichment analysis

## Abstract

Cold hypersensitivity in hands and feet (CHHF) affects 20–52% of East Asian populations, is more prevalent in women, and is managed with vasodilators in Western medicine or herbal therapies in traditional East Asian medicine. This study was conducted to compare the pharmacological characteristics of two heat-clearing (HCHs, Scutellariae Radix and Coptidis Rhizoma) and two blood-tonifying (BTHs, Angelicae Sinensis Radix and Paeoniae Radix Alba) herbs used for CHHF. Candidate herbs were selected based on herbology classifications, PubMed searches, and the Korean Medicine Clinical Practice Guideline for CHHF. A total of 61 active compounds and 323 corresponding protein targets with CHHF were retrieved from the traditional Chinese medicine systems pharmacology database and analysis platform (TCMSP), standardized via UniProt, and intersected with CHHF-related genes from GeneCards. Protein–protein interaction networks were constructed, core targets identified, and functional enrichment analyses performed using Reactome databases. A total of 166 herb–CHHF common targets were identified, including 24 shared across all four herbs. HCHs were primarily enriched in hemostasis-, immune-, and signaling-related pathways, whereas BTHs were associated with immune regulation, metabolism, and neuronal modulation. Notably, Scutellariae Radix and Paeoniae Radix Alba showed substantial overlap in enriched pathways. These findings suggest that HCHs and BTHs may be associated with distinct yet potentially relevant molecular mechanisms in CHHF. However, because the present analysis was conducted at the single-herb level, these findings cannot be directly generalized to the clinical use of multi-herb formulas and should be interpreted as hypothesis-generating.

## 1. Introduction

Cold hypersensitivity is a condition in which specific parts of the body feel unusually cold and painful, even at temperatures that would not normally provoke such sensations, making it difficult to maintain daily activities [1]. Cold hypersensitivity primarily arises from impaired blood circulation and reduced heat production, but it can also be induced by a wide range of underlying factors. Common contributors include decreased physical strength due to gastrointestinal disturbance, anemia, hypotension, capillary constriction caused by dysautonomia, pelvic congestion, and disturbances in body fluid metabolism. Various disorders—such as Raynaud’s phenomenon (RP), peripheral neuritis, polyneuropathy, and carpal tunnel syndrome—can also elicit cold hypersensitivity. CHHF is generally defined as a subjective sensation of coldness in the hands or feet, and it may present as coldness limited to the hands, to the feet, or involving both the hands and feet [2]. This condition is commonly observed in approximately 20–52% of East Asian populations, and a twin-based genetic study conducted in Korea reported a female-to-male ratio of 3:2 in CHHF prevalence, indicating increased rates among women [3]. According to the Clinical Practice Guideline of Korean Medicine for Cold Hypersensitivity in Hands and Feet (hereafter CPG of CHHF), this condition is not classified as an independent disease entity. CHHF is defined as a clinical category that includes primary RP and other peripheral cold-related disorders, while excluding secondary RP caused by identifiable underlying diseases [1]. Therefore, CHHF is not synonymous with RP but represents a broader clinical spectrum, within which primary RP is considered a subtype. Although research articles and reviews related to CHHF have been steadily increasing, a clear and universally accepted definition of CHHF has not yet been established [4].

In Western medicine, CHHF is regarded as one of the representative clinical manifestations of RP, and most patients with CHHF are prescribed antihypertensive or vasodilatory agents [4]. Traditional East Asian medicine—including Traditional Chinese Medicine (TCM), KM, and Japanese Kampo Medicine—interprets “Cold (冷)” as a major pathogen [4]. From the perspective of Yin–Yang theory, depletion of Yang-qi and predominance of Yin-qi result in a decline in body temperature [4]. Consequently, traditional East Asian medical systems emphasize a holistic and individualized approach aimed at restoring the balance of Yin and Yang as a central therapeutic strategy for CHHF [4]. In Korean Medicine (KM), cold hypersensitivity is understood to result from disruptions in the circulation of qi and blood or deficiencies of qi, blood, and yang due to pathological patterns such as spleen-yang deficiency, kidney-yang deficiency, blood stasis, blood deficiency, qi deficiency, or water poisoning [2]. Previous studies have suggested that herbal medicines rooted in traditional East Asian medicine have the potential to serve as safe and effective treatment options for CHHF [4].

Unlike conventional pharmaceuticals, which typically contain a single active ingredient designed to target a specific molecular pathway, herbal medicines consist of multiple bioactive compounds and are known to modulate various biological targets simultaneously [5]. In other words, the pharmacologically active compounds of medicinal herbs exert broad therapeutic effects through various pathways, while showing minimal adverse reactions in the human body [6]. Herbal medicines are categorized into several groups, among which Heat-Clearing Herbs (淸熱藥, HCHs) represent one major category. Most herbs within this group possess a cold nature (寒性) and function to eliminate heat, drain pathogenic fire (火), dry dampness, cool the blood, and detoxify harmful substances. Their primary therapeutic action is to dispel internal heat. In this context, the concept of “heat” or “fire” corresponds to fever associated with inflammatory conditions [7]. Accordingly, HCHs are currently recognized and utilized for their anti-inflammatory and inflammation-modulating properties.

According to the study by Chang et al. [8], the pathogenesis of CHHF may involve increased sympathetic activity and exaggerated dysfunction of endothelial cells (ECs). A study has reported that herbal medicines exert effects such as modulation of sympathetic nervous system activity [9]. While CHHF has traditionally been classified as a cold pattern (寒證) and treated mainly with warming methods (溫法) using interior-warming herbs [2], modern pharmacological studies on representative HCHs and their constituents have suggested modulation of sympathetic activity, along with anti-inflammatory and vascular effects [10]. Traditionally, HCHs have been used to drain (瀉) pathogenic “heat (熱)” from the body. Accordingly, this study explored the potential mechanisms underlying HCHs using a network pharmacology approach grounded in these conceptual perspectives.

In RP, CHHF is attributed to recurrent and reversible excessive vasoconstriction of peripheral vessels, impaired blood flow, and endothelial dysfunction [10].

Endothelial dysfunction involves key vasoconstrictive mediators such as endothelin-1, angiotensin II, and angiopoietin-2. Endothelin-1 is a major endothelium-derived vasoconstrictor, and patients with RP exhibit reduced endothelium-dependent vasodilation, which contributes to digital ischemia [10]. The expression of these vasoconstrictors has been reported to be upregulated by pro-inflammatory cytokines, including tumor necrosis factor-α (TNF-α) and interferon-γ (IFN-γ) [11]. This suggests that CHHF is closely related not only to vascular dysfunction but also to inflammatory responses.

The pathophysiology of endothelial dysfunction is further associated with p38 mitogen-activated protein kinase (p38 MAPK) activation [12].

Recent studies suggest that microvascular stabilization mechanisms, including endothelial cell (EC)–pericyte interactions, play an important role in maintaining peripheral blood flow. CXCR3–CXCL11 signaling has been implicated in regulating EC proliferation and pericyte recruitment [13].

In addition, dysautonomia-related CHHF has been associated with sympathetic overactivity, in which α2-adrenergic receptors (α2-ARs), particularly the α2A-AR subtype, mediate rapid vasoconstriction in cutaneous vessels [8,14,15].

Herbal medicines consist of complex mixtures of compounds that act on multiple pathological targets and pathways. Conventional pharmacological approaches therefore have limitations in elucidating their mechanisms of action [16]. In 2013, Shao Li proposed the concept of “network pharmacology,” offering a new strategy for uncovering the mechanistic basis of herbal formulas [17]. Network analysis and network pharmacology utilize database mining and bioinformatic computation to analyze and simulate human protein–protein interaction (PPI) networks, thereby revealing the complex interaction patterns between drugs and diseases [18].

Network pharmacology has increasingly been applied to investigate the mechanisms underlying the therapeutic effects of herbal medicines in various diseases [19], and this approach is particularly suited to herbal medicine research because it enables the systematic analysis of multiple components, pathways, and targets [19,20].

## 2. Materials and Methods

**Figure 1:**
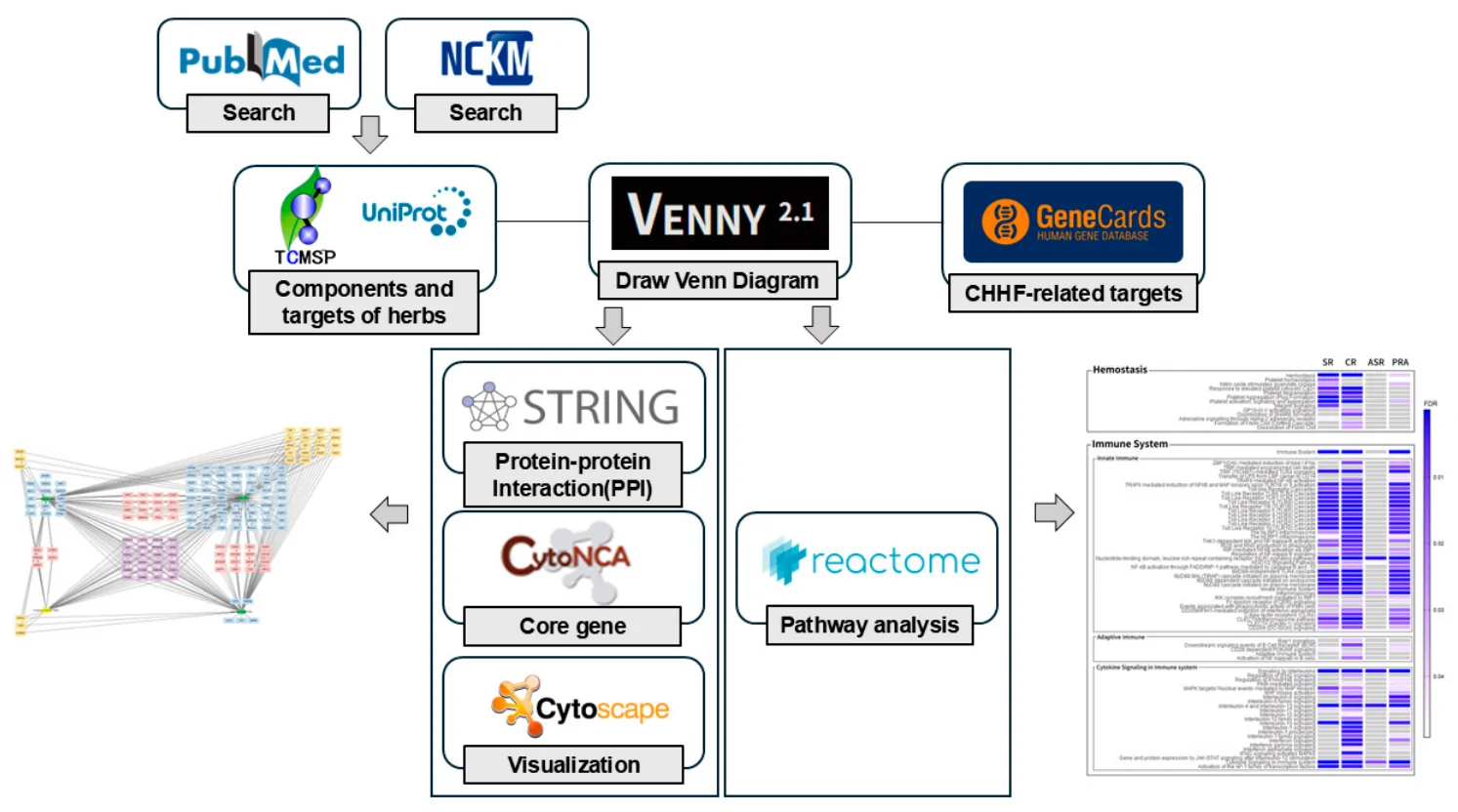
Schematic workflow of the network pharmacology and pathway analysis.

Herbs were chosen by literature screening (PubMed, National Clearinghouse for Korean Medicine (NCKM)). Herb compounds and targets (TCMSP) were standardized (UniProt) and intersected with CHHF targets (GeneCards) using Venny 2.1. Protein–protein interaction (PPI) networks (search tool for the retrieval of interacting genes/proteins (STRING)), core-gene analysis (Cytoscape network centrality analysis (CytoNCA 2.1.6)), visualization (Cytoscape 3.10.3), and pathway enrichment analysis (Reactome) were performed. The overall workflow of the network pharmacology and pathway enrichment analyses is illustrated in Figure 1.

### 2.1. Selection of Herbal Medicines for Analysis

#### 2.1.1. Selection of Heat-Clearing Herbs: PubMed

In this study, herbal medicines classified as “Heat-Clearing Herbs” in *Herbology* [21] were selected as the target group for analysis. Candidate herbs were identified through a literature search of the PubMed database (https://pubmed.ncbi.nlm.nih.gov/ (accessed on 2 August 2025)). Searches were conducted by combining the English names of the herbs with clinical and pathophysiological terms related to CHHF, including “primary Raynaud’s phenomenon”, “peripheral mechanism”, “increased vasoconstriction”, “sensitive to cold”, and “hot flushes” using the OR operator.

This search strategy was based on the fact that CHHF is not typically reported as an independent disease entity but rather described under various diagnoses or clinical/pathophysiological terms such as primary RP or other specified peripheral vascular disorders.

Accordingly, key symptoms associated with CHHF were identified through a review of the relevant academic literature, and these were used as search terms. Based on the search outcomes, the two most frequently appearing herbs were selected as the final target herbs for this study.

#### 2.1.2. Selection of Commonly Used Herbal Medicines for CHHF: Clinical Practice Guideline of Korean Medicine—CHHF Figures, Tables, and Schemes

Clinical practice guidelines (CPGs) are evidence-based documents developed through systematic and scientific processes to assist healthcare providers and patients in making informed decisions throughout medical care [2].

To identify herbal medicines commonly used in clinical practice for CHHF, we collected prescriptions recommended in the Clinical Practice Guideline of Korean Medicine for Cold Hypersensitivity in Hands and Feet (hereafter CPG of CHHF). The CPG of CHHF is available from the NCKM (https://nikom.or.kr/nckm/index.do (accessed on 2 August 2025)). Prescriptions with an evidence-based recommendation grade of C or higher, or a consensus-based recommendation grade of GPP, were included.

Subsequently, all constituent herbs from the prescriptions listed in the CPG of CHHF were extracted. The names of herbal medicines were standardized based on *Herbology* [21]. According to this text, “Peony root (芍藥, Paeoniae Radix)” is not listed as an independent item; instead, it is categorized either as White Peony root (白芍藥, Paeniae Radix Alba) under blood-tonifying herbs (補血藥, BTHs) or as Red Peony root (赤芍藥, Paeoniae Radix Rubra) under heat-clearing and blood-cooling herbs (淸熱凉血藥). For each prescription, the source was therefore reviewed to determine whether Peony root referred to White or Red Peony root.

Additionally, because “Red Poria (赤茯苓; in Korea, the light reddish portion beneath the outer peel (皮層) is referred to as 赤茯苓)” is not listed separately in the *Herbology* table of contents, it was integrated into “Poria (茯苓, Poria Sclerotium).” Although Korean Red Ginseng (紅蔘, Ginseng Radix Rubra, steamed and dried ginseng prepared after removing the fine rootlets (鬚根) of Ginseng Radix) is not listed as a separate item in *Herbology*, it was not merged with Ginseng (人蔘), as the CPG of CHHF identifies “Korean Red Ginseng granule” as distinct from Ginseng used in other prescriptions.

The standardized herbal names were then sorted by frequency, and the top two herbs were selected as the final study targets. Licorice (甘草, Glycyrrhizae Radix et Rhizoma) was excluded because it is used extremely broadly as a harmonizing herb (調和諸藥); it appears in 70 out of the 113 prescriptions in the Shanghanlun (傷寒論) [22].

### 2.2. Collection of Active Compounds and Target Gene/Protein Data: TCMSP

Data on the active compounds and target gene/protein information of the selected herbal medicines were collected through searches in the TCMSP (https://www.tcmsp-e.com/ (accessed on 2 August 2025)). TCMSP is a systems pharmacology platform that provides information about herbal ingredients, their corresponding molecular targets, and associated diseases. It contains data on 499 herbs, 29,384 compounds, and 3311 target proteins, as well as 12 key ADME (absorption, distribution, metabolism, and excretion) characteristics that describe the pharmacokinetic properties of each compound [23].

According to previous research, approximately one-third of studies using TCMSP apply compound-selection criteria of oral bioavailability (OB) ≥ 30% and drug-likeness (DL) ≥ 0.18 [24]. In the present study, active compounds were therefore selected based on these same criteria.

Next, the “Target Name” corresponding to each active compound was collected from the TCMSP database. To standardize protein names for the target proteins associated with the active compounds, each Target Name obtained from TCMSP was searched in the UniProt database (Database of Protein Sequences, https://www.uniprot.org/ (accessed on 2 August 2025)). Only entries with a status of “Reviewed (Swiss-Prot)” and classified under “Human” in the Popular Organisms’ category were included. From the resulting matches, proteins whose Protein Names exactly corresponded to the queried Target Name were selected, and their Entry (UniProt ID) and Gene Names were retrieved.

### 2.3. Collection of Target Gene/Protein Data for CHHF: GeneCards

Target genes associated with CHHF were collected by searching the human gene database GeneCards (https://www.genecards.org/ (accessed on 2 August 2025)) using the keyword “cold hypersensitivity in hands and feet.” Protein entries without UniProt IDs and duplicated data were excluded from the dataset.

### 2.4. Network Pharmacology Analysis

#### 2.4.1. Collection of Common Herb–CHHF Target Proteins: Venny, Cytoscape

To identify the common target proteins shared between each herbal medicine and CHHF, Venny 2.1.0 (https://bioinfogp.cnb.csic.es/tools/venny/ (accessed on 15 August 2025)) was first employed. All datasets were entered in UniProt ID format.

The overlapping target proteins between each herb and CHHF were then re-entered into Venny to examine the distribution and intersection patterns of proteins commonly targeted by the four herbs—Scutellariae Radix (黃芩), Coptidis Rhizoma (黃連), Angelicae Sinensis Radix (當歸), and Paeoniae Radix Alba (白芍藥).

The resulting common-target protein network was visualized using Cytoscape 3.10.3, an open-source software platform designed for analyzing, integrating, and visualizing biological interaction networks.

#### 2.4.2. Protein–Protein Interaction (PPI) Network Analysis: STRING

Based on the common target proteins identified between each herbal medicine and CHHF using Venny 2.1.0, a PPI network analysis was conducted to explore the potential pharmacological mechanisms of the selected herbs.

The common target proteins were entered into the STRING 12.0 database (https://www.string-db.org/ (accessed on 27 August 2025)) in UniProt ID format to generate the PPI network. During this process, the organism was restricted to *Homo sapiens*, and the confidence threshold was set at a confidence score ≥ 0.7 (high confidence). All other parameters were maintained at their default settings.

#### 2.4.3. Extraction of Core Genes

The structural characteristics of the PPI network were analyzed using PPI analysis results. The CytoNCA 2.1.6 plugin in Cytoscape 3.10.3 was used to calculate the topological properties of each node. Four key centrality parameters—betweenness (BC), closeness (CC), degree (DC), and eigenvector centrality (EC)—were applied.

The topological indices for each node were exported from Cytoscape as a table; then, Python 3.12 was used to determine and extract the core genes. Following the approach commonly used in previous network pharmacology studies [25,26,27,28,29], genes exceeding the median value across all four centrality indices (BC, CC, DC, and EC) were defined as core genes. Because each index reflects a distinct aspect of node importance within the network, this intersection-based approach was intended to minimize bias arising from reliance on a single index and to select only nodes that were consistently important across multiple topological criteria. The selection criteria for core genes for each herbal medicine are summarized in Table 1.

#### 2.4.4. Pathway Enrichment Analysis: Reactome (Home–Reactome Pathway Database)

To explore the molecular interaction pathways involved in the pathophysiology of CHHF, the common target proteins shared between each herbal medicine and CHHF—identified previously using Venny 2.1.0—were entered into the Reactome database (version 93; https://reactome.org/ (accessed on 25 August 2025)). Reactome is an open-source, open-access pathway database that provides biologically curated pathway information constructed through expert manual annotation and peer review [30].

For each herbal medicine, the common target proteins associated with CHHF were uploaded to Reactome in UniProt ID format to perform biological pathway enrichment analysis. From the resulting output, only the pathway identifier, pathway name, and false discovery rate (FDR) values of entities were extracted. The pathway identifier was used to classify each pathway according to its higher-level category.

After compiling the pathway names and FDR values of entities for all four herbs, only pathways with FDR values of entities < 0.05 were retained. The Reactome database contains 29 top-level pathway categories, among which five categories were selected for analysis based on their relevance to the known pathophysiology of CHHF.

In patients with RP, abnormalities in the coagulation system—such as platelet activation, increased blood viscosity, and microthrombus formation—have been observed [31], and these factors can be considered to be associated with the impaired blood flow observed in CHHF, which represents a clinical manifestation of RP. In addition, it has been reported that immune responses may partially contribute to the pathophysiology of RP [10]. Cold-induced vasoconstriction is mediated by redox signaling in vascular smooth muscle cells (VSMCs), during which mitochondrial ROS stimulate the RhoA/Rho kinase signaling pathway. This activation subsequently promotes the translocation of α2C-adrenergic receptors (α2C-ARs) to the cell surface, leading to vasoconstriction, a mechanism closely associated with CHHF [32].

Additionally, from a neurogenic perspective, exposure to cold or stress increases sympathetic nervous system activity, resulting in the release of norepinephrine, which activates postsynaptic α-adrenergic receptors on vascular smooth muscle and induces vasoconstriction, ultimately contributing to CHHF [33].

Repeated cycles of ischemia–reperfusion in RP have also been shown to generate excessive ROS, causing metabolic stress in affected tissues [33]. Based on these cumulative findings, five top-level pathway categories highly relevant to CHHF pathophysiology—Hemostasis, Immune System, Signal Transduction, Neuronal System, and Metabolism—were selected as the focus of the present analysis.

## 3. Results

### 3.1. Selection of Herbal Medicines

#### 3.1.1. Selection of Heat-Clearing Herbs (淸熱藥): PubMed Search

According to the 『Herbology』 [21], a total of 52 herbal medicines are classified under the category of “heat-clearing herbs.” Each of these herbs was searched in PubMed using the following query: “(Herb name) AND ((primary Raynaud’s phenomenon) OR (peripheral mechanism) OR (increased vasoconstriction) OR (sensitivity to cold) OR (hot flushes)).”

The number of publications retrieved for each herb was summarized (Table 2), and Scutellariae Radix and Coptidis Rhizoma were ultimately selected as the target herbs for this study.

#### 3.1.2. Selection of Commonly Used Herbal Medicines for CHHF

Based on the CPG of CHHF, a total of twelve prescriptions are recommended (with evidence-based recommendation grade ≥ C or consensus-based grade GPP) [2].

The first-line prescriptions include Dangguisayeoktang (當歸四逆湯), Dangguisayeokgaosuyusaengangtang (當歸四逆加吳茱萸生薑湯), Ongyeongtang (溫經湯), Korean Red Ginseng (紅蔘) granules, Gyejibokryeonghwan (桂枝茯苓丸), and Dangguijakyaksan (當歸芍藥散) [2].

In addition, Yijungtang (理中湯), Bojungigkitang (補中益氣湯), Sipjeondaebotang (十全大補湯), Ojeoksan (五積散), Palmijihwanghwan (八味地黃丸), and Gyejitang (桂枝湯) are recommended for use according to syndrome differentiation, based on formal expert consensus [2].

An analysis of these twelve prescriptions identified 37 constituent herbs. When sorted by frequency of occurrence, Licorice (甘草, Glycyrrhizae Radix et Rhizoma), White Peony root (白芍藥, Paeoniae Radix Alba), and *Angelica gigas* root (當歸, Angelicae Gigantis Radix) appeared in seven of the formulas. Considering the harmonizing property of Licorice (調和諸藥), it was excluded from the study. Consequently, Paeoniae Radix Alba and Angelicae Gigantis Radix were selected as the final target herbs.

### 3.2. Collection of Active Compounds and Target Proteins: TCMSP

Active compounds were retrieved from the TCMSP based on the criteria of OB ≥ 30% and DL ≥ 0.18.

The corresponding target genes and proteins related to these active compounds were also collected, and the results are summarized in Table 3. The active compounds are also listed in Table A1, Table A2, Table A3 and Table A4. Their biological activities and key findings are reported in Table A5.

In the case of Angelicae Gigantis Radix (*Angelica gigas* root, Danggui), the Korean Pharmacopoeia defines it as the root of *Angelica gigas Nakai* (Korean Danggui), whereas the Chinese Pharmacopoeia defines Danggui as the root of *Angelica sinensis* (Oliv.) Diels (Chinese Danggui). As only Chinese Danggui is available in the TCMSP database, the pharmacognostic name Angelicae Sinensis Radix was used in this study.

Subsequently, UniProt IDs of the target proteins were collected.

For several proteins that could not be precisely identified in the UniProt database, their UniProt IDs were determined through consensus among three independent researchers (Table 4).

### 3.3. Collection of Target Proteins Related to CHHF: GeneCards

Target genes and proteins associated with CHHF in hands and feet were retrieved from the GeneCards database using the search term “cold hypersensitivity in hands and feet.”

Following the exclusion of entries without UniProt IDs and the removal of duplicates, a total of 2588 unique target proteins were finalized for further analysis starting from an initial pool of 2750 identified proteins.

### 3.4. Network Pharmacology Analysis

#### 3.4.1. Identification of Common Target Proteins Between Herbal Medicines and CHHF: Venny

Common target proteins shared between the selected herbal medicines and CHHF were identified using Venny 2.1.0.

**Figure 2:**
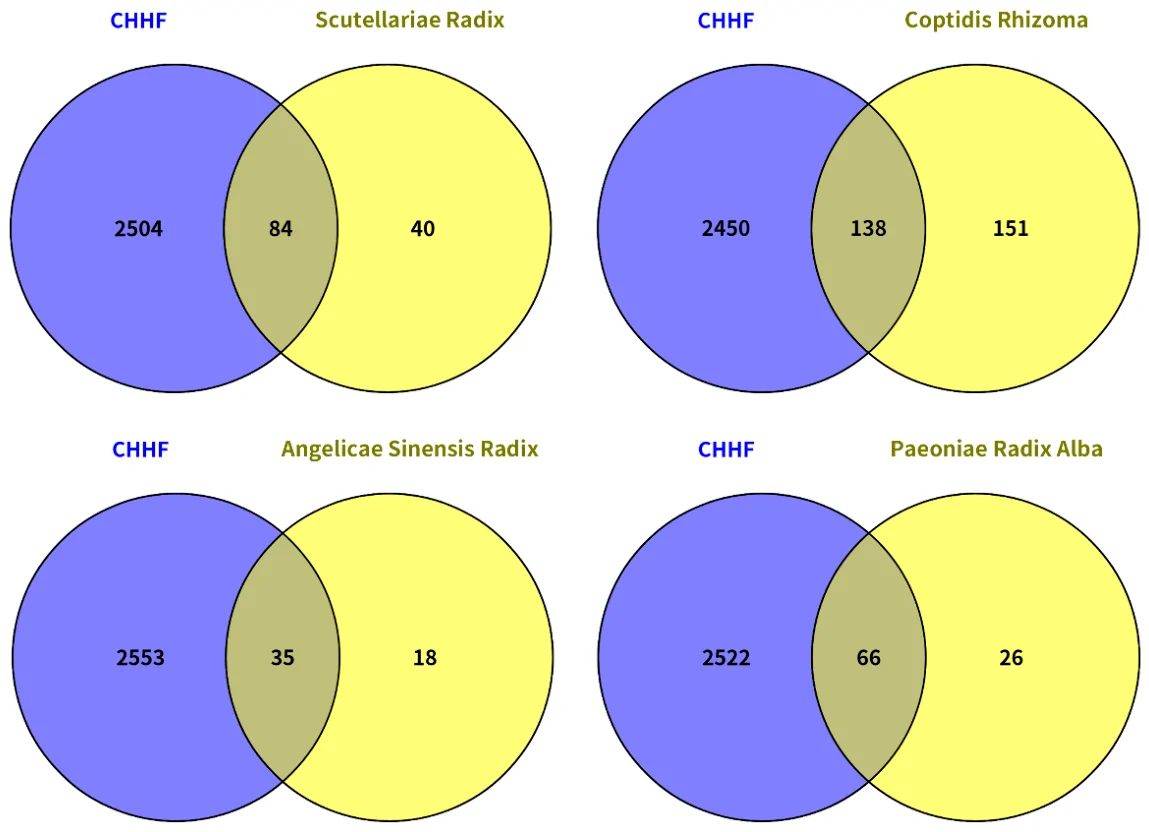
Venn diagrams showing overlapping targets between CHHF and selected herbs.

As a result, 84, 138, 66, and 35 overlapping target proteins were identified for Scutellariae Radix, Coptidis Rhizoma, Paeoniae Radix Alba, and Angelicae Sinensis Radix, respectively (Figure 2 and Table 5).

**Figure 3:**
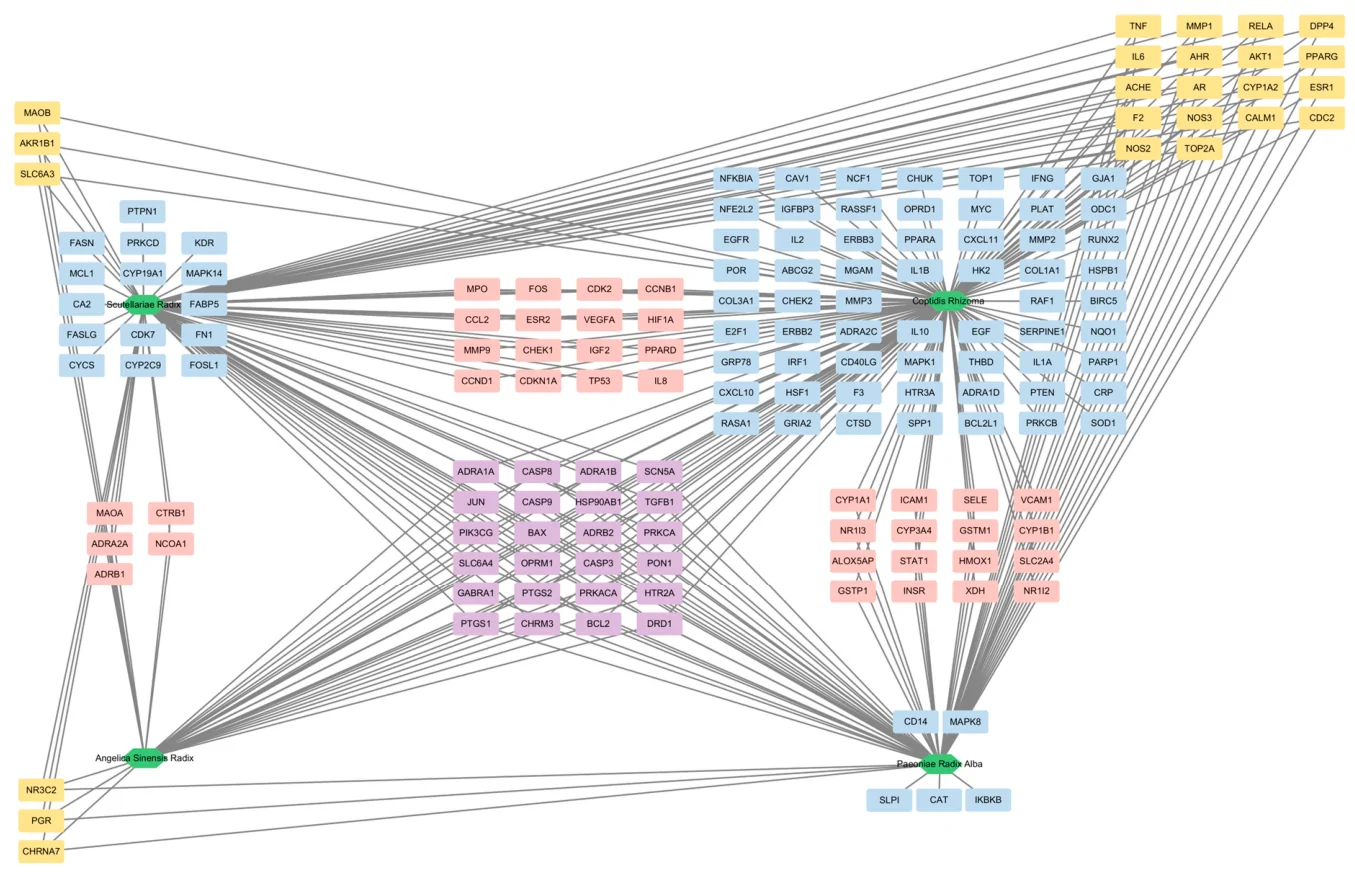
Network of overlapping targets between CHHF and four selected herbs visualized by Cytoscape. Green hexagons: four herbal medicines—Scutellariae Radix, Coptidis Rhizoma, Angelicae Sinensis Radix, and Paeoniae Radix Alba; Light blue squares: targets unique to a single herb; Pink squares: proteins targeted by two herbs; Yellow squares: proteins commonly targeted by three herbs; Purple squares: proteins shared by all four herbs.

The common target proteins shared between the herbal medicines and CHHF were reanalyzed using Venny, and the distribution and overlapping patterns of target proteins among the four herbal medicines were visualized with Cytoscape (Figure 3). Overlapping target proteins between CHHF and the four selected herbs are also listed in Table A6.

In the network diagram, four green hexagons represent the four herbal medicines—Scutellariae Radix, Coptidis Rhizoma, Angelicae Sinensis Radix, and Paeoniae Radix Alba. Light blue squares indicate targets unique to a single herb, while pink squares represent proteins targeted by two. Yellow squares denote proteins commonly targeted by three herbs, and purple squares correspond to proteins shared by all four.

A total of 166 target proteins were identified from the four herbs, of which 85 were targeted by at least two herbs. In contrast, 81 proteins were uniquely targeted by a single herb, with Coptidis Rhizoma accounting for the largest proportion (61 unique targets). Notably, Angelicae Sinensis Radix had no unique targets. A total of 16 proteins were co-targeted exclusively by the two HCHs (Scutellariae Radix and Coptidis Rhizoma), whereas no targets were shared solely by the two BTHs (Angelicae Sinensis Radix and Paeoniae Radix Alba). A total of 18 proteins were co-targeted by Scutellariae Radix, Coptidis Rhizoma, and Paeoniae Radix Alba, and 24 were found to be common targets of all four herbs.

#### 3.4.2. Protein–Protein Interaction (PPI) Network Analysis: STRING

**Figure 4:**
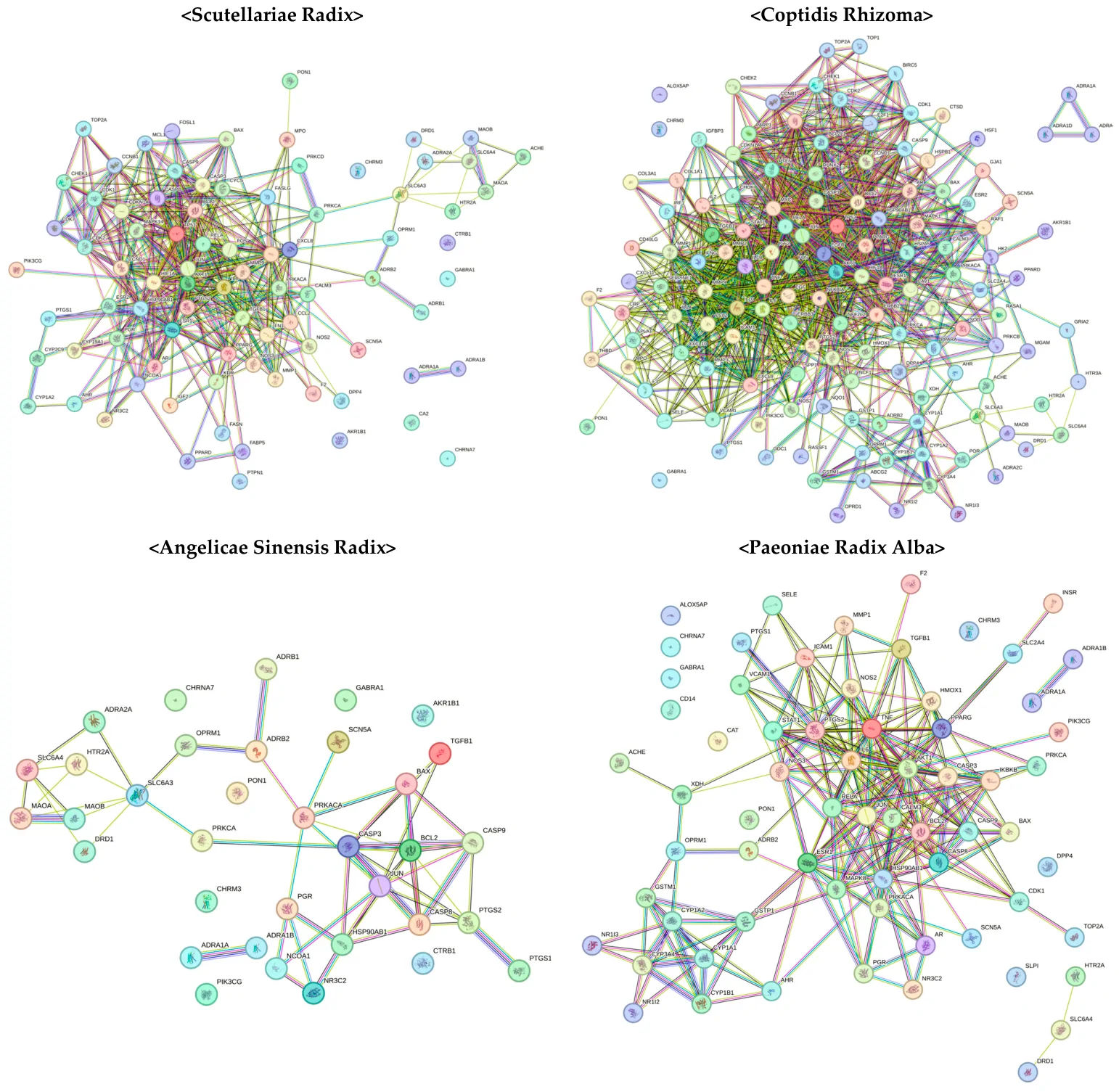
Visualization of the common target genes and proteins of the four herbs (Scutellariae Radix, Coptidis Rhizoma, Angelicae Sinensis Radix, and Paeoniae Radix Alba). STRING 12.0 software was used to create the PPI network.

The common target genes and proteins of the four herbs identified through Venny 2.1.0 were entered into the STRING 12.0 database to construct individual PPI networks for each herb (Figure 4).

Among them, Coptidis Rhizoma exhibited the highest average node degree (16.5), indicating the most extensive PPI compared to the other herbs. In contrast, Angelicae Sinensis Radix showed the lowest average node degree (3.2), suggesting relatively limited interactions. Scutellariae Radix and Paeoniae Radix Alba displayed intermediate levels, with average node degrees of 10.6 and 7.03, respectively (Table 6).

#### 3.4.3. Identification of Core Genes

Nodes with BC, CC, DC, and EC values higher than the median were defined as core genes.

**Figure 5:**
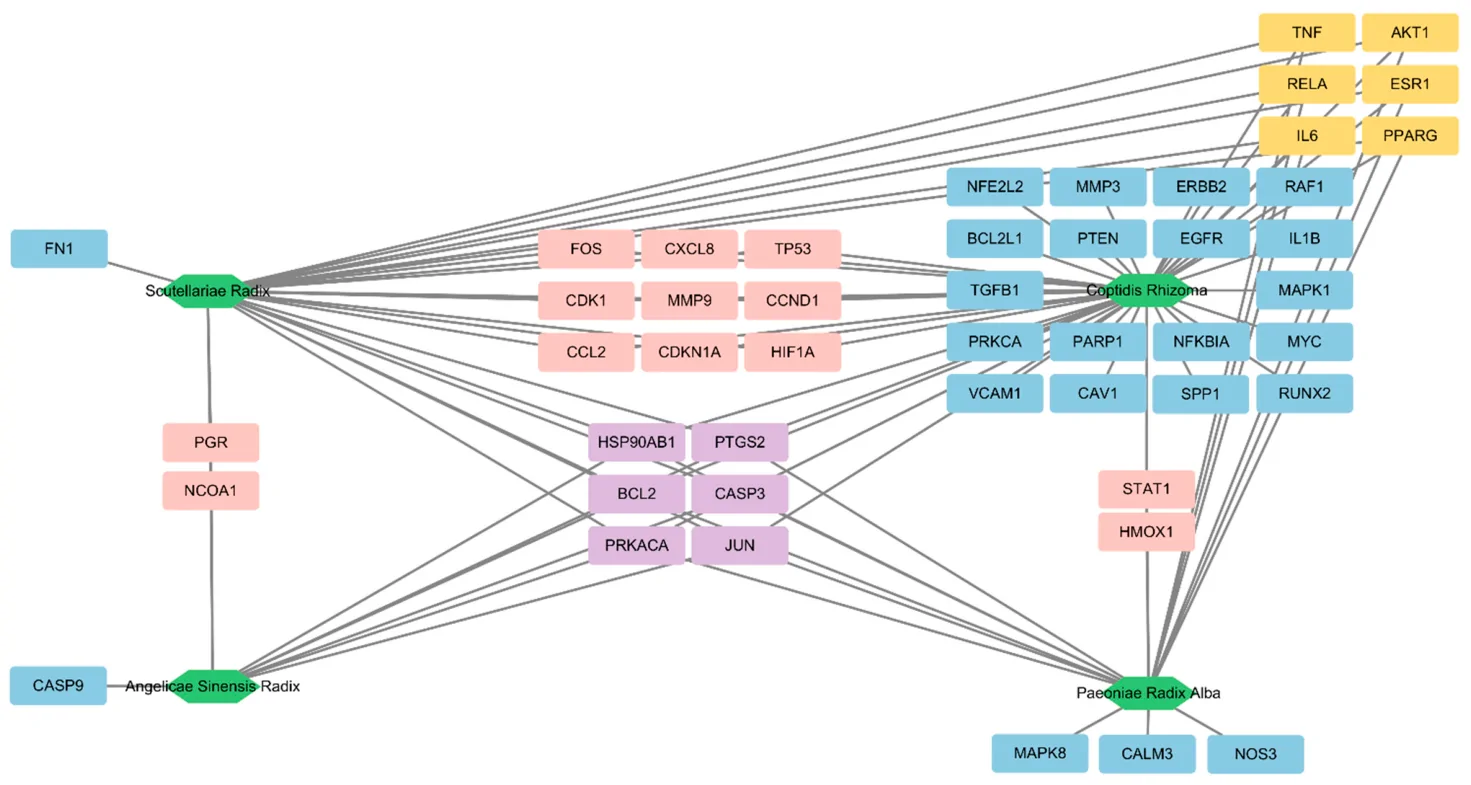
Network of overlapping core genes between CHHF and four selected herbs (Scutellariae Radix, Coptidis Rhizoma, Angelicae Sinensis Radix, and Paeoniae Radix Alba) visualized by Cytoscape. Green hexagons: four herbal medicines—Scutellariae Radix, Coptidis Rhizoma, Angelicae Sinensis Radix, and Paeoniae Radix Alba; Light blue squares: targets unique to a single herb; Pink squares: proteins targeted by two herbs; Yellow squares: proteins commonly targeted by three herbs; Purple squares: proteins shared by all four herbs.

The numbers of core genes identified for each herbal medicine are summarized in Table 6, and the corresponding protein interaction networks constructed based on these core genes are illustrated in Figure 5. Overlapping core genes between CHHF and the four selected herbs are also listed in Table A7.

#### 3.4.4. Pathway Enrichment Analysis: Reactome

The common target genes and proteins between each herbal medicine and CHHF were input into the Reactome Pathway Database (Home–Reactome Pathway Database) in UniProt ID format.

Among the biological pathways identified, only those with FDR values of entities < 0.05 were retained, resulting in 494 pathways belonging to 19 supercategories.

Of these, 233 pathways corresponding to the five major supercategories considered most relevant to CHHF—Hemostasis, Immune System, Signal Transduction, Neuronal System, and Metabolism—were selected for further analysis.

The number of pathways significantly associated with CHHF for each herbal medicine was as follows: Scutellariae Radix (153), Coptidis Rhizoma (188), Angelicae Sinensis Radix (44), and Paeoniae Radix Alba (144).

**Figure 6:**
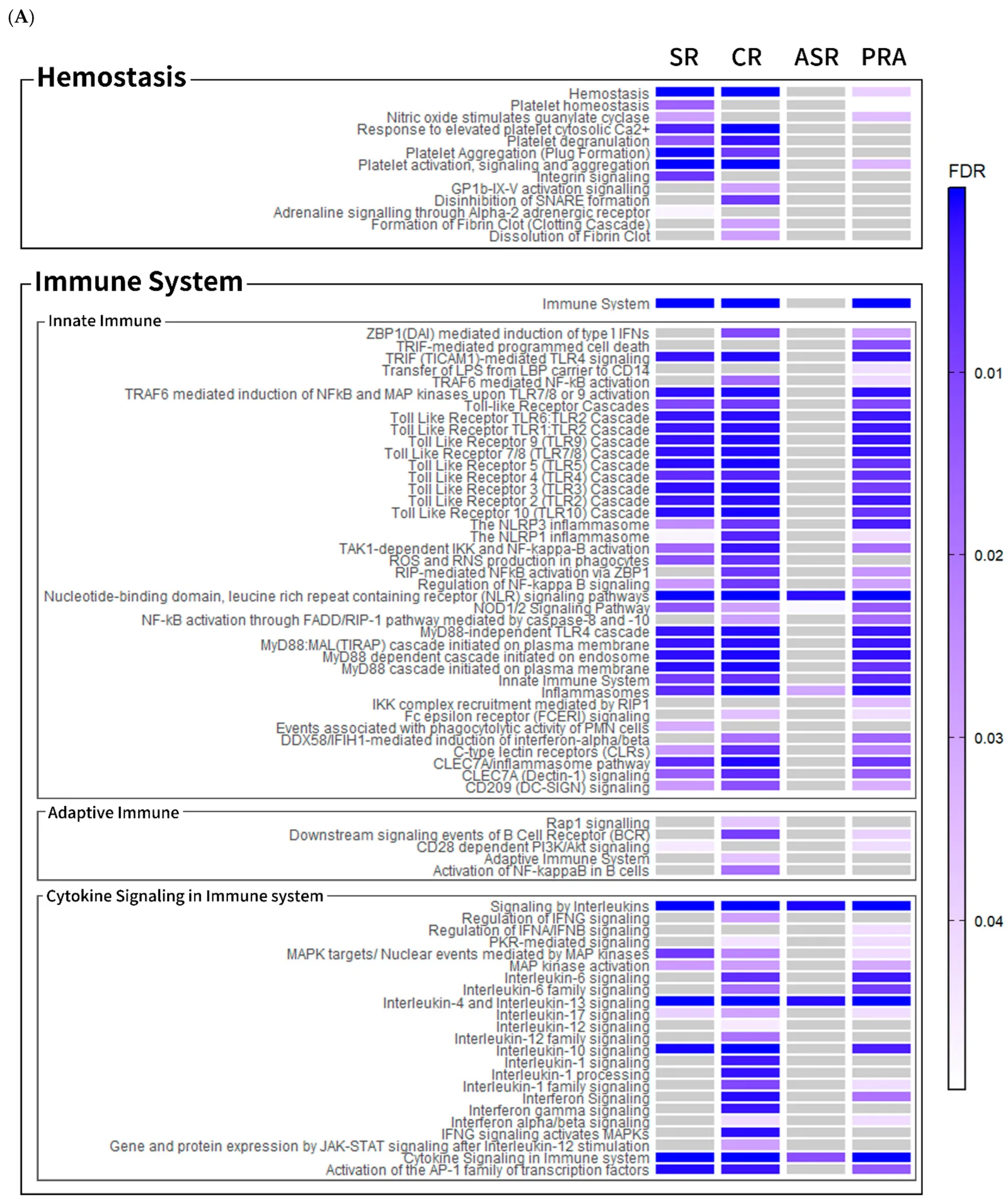

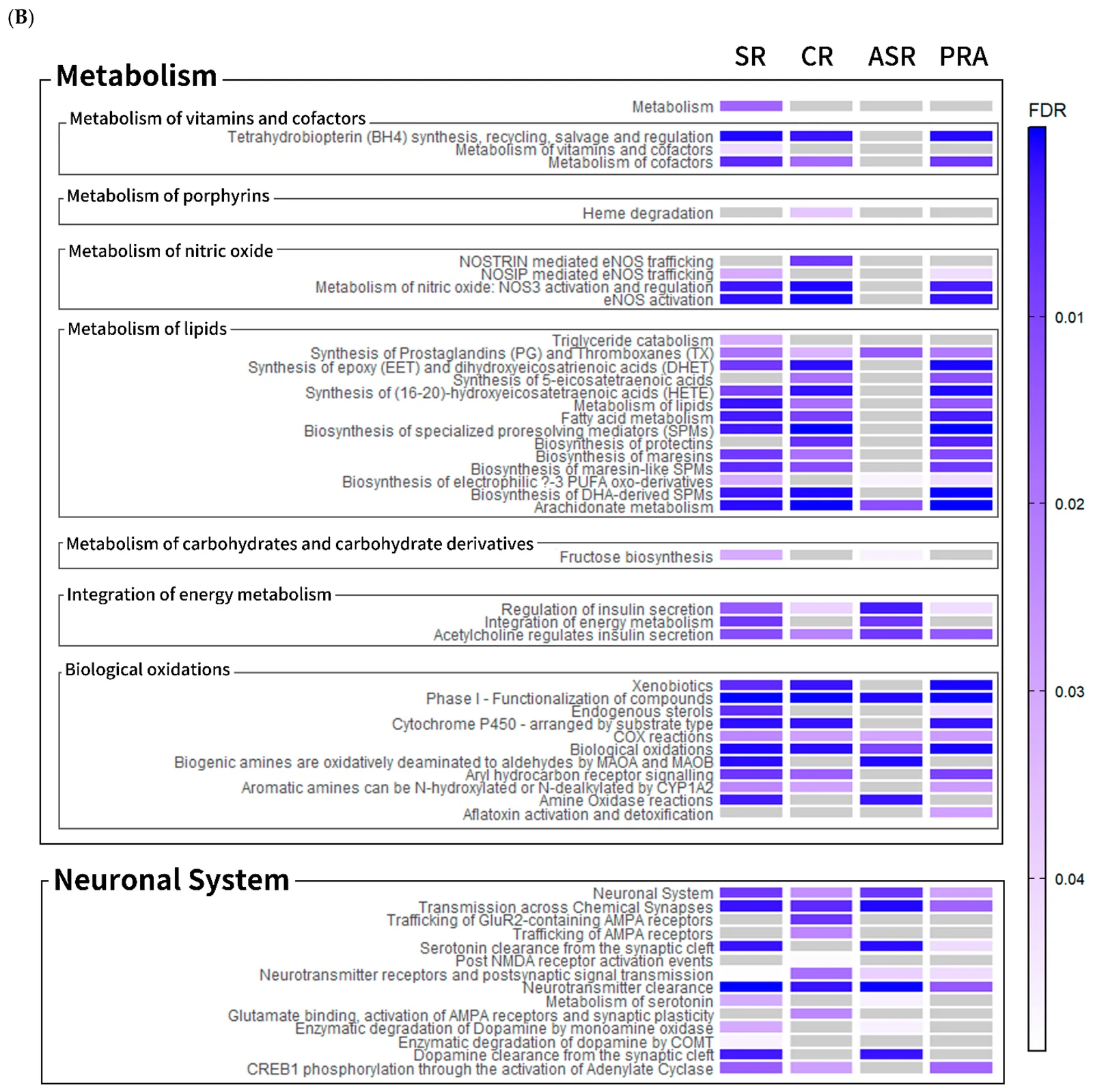

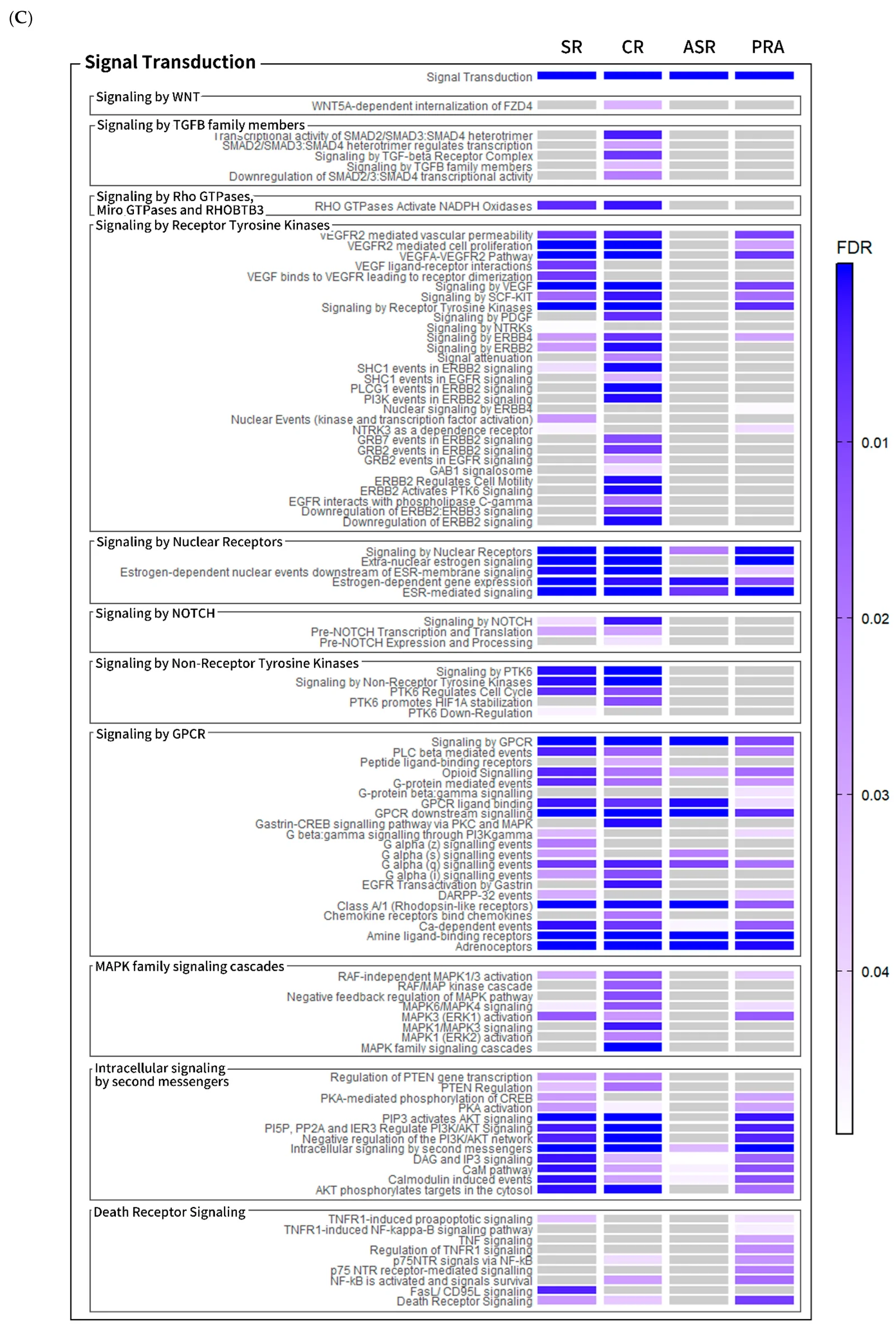
Differences in molecular pathways among SR, CR, ASR, and PRA. SR: Scutellariae Radix; CR: Coptidis Rhizoma; ASR: Angelicae Sinensis Radix; PRA: Paeoniae Radix Alba. (**A**) Hemostasis, immune system-related molecular pathways. (**B**) Metabolism, neuronal system-related molecular pathways. (**C**) Signal transduction-related molecular pathways.

The number of pathways in each supercategory for each herb is summarized in Table 7, and the detailed distribution of enriched pathways is presented in Figure 6A–C.

Comparison of shared pathways among the four herbs revealed that Scutellariae Radix and Coptidis Rhizoma shared 119 pathways; Scutellariae Radix and Angelicae Sinensis Radix, 44 pathways; Scutellariae Radix and Paeoniae Radix Alba, 116 pathways; Coptidis Rhizoma and Angelicae Sinensis Radix, 34 pathways; Coptidis Rhizoma and Paeoniae Radix Alba, 12 pathways; and Angelicae Sinensis Radix and Paeoniae Radix Alba, 36 pathways.

Notably, among the 116 pathways shared by Scutellariae Radix and Paeoniae Radix Alba, approximately 76% of those of the former and 80% of those of the latter overlapped, indicating a close mechanistic association between these two herbs in CHHF treatment.

All 44 pathways identified for Angelicae Sinensis Radix were shared with Scutellariae Radix, suggesting that the mechanisms of the former are largely encompassed within those of the latter.

A total of 34 pathways were found to be common to all four herbs.

When examined by supercategory, the number of significant pathways related to Hemostasis was nine for Scutellariae Radix, nine for Coptidis Rhizoma, four for Paeoniae Radix Alba, and null for Angelicae Sinensis Radix.

Among these, pathways directly associated with platelet activation and aggregation, integrin signaling, and fibrin clot formation were observed exclusively for Scutellariae Radix and Coptidis Rhizoma.

This finding suggests that Scutellariae Radix, while sharing mechanistic similarities with Paeoniae Radix Alba, also exhibits unique pathways related to blood coagulation and hemostasis in conjunction with Coptidis Rhizoma.

Regarding the proportions of pathways in the Immune System and Signal Transduction categories, Coptidis Rhizoma accounted for 76%, Paeoniae Radix Alba for 73%, Scutellariae Radix for 66%, and Angelicae Sinensis Radix for 55%.

Among them, Angelicae Sinensis Radix showed the lowest proportion of Immune System-related pathways (14%) but exhibited the highest proportions of Metabolism (27%) and Neuronal System (18%) pathways, indicating its relative emphasis on metabolic and neuroregulatory mechanisms compared with the other herbs.

## 4. Discussion

KM has played a significant role in health management and disease treatment across East Asia for over two millennia. Herbal medicine has been widely utilized for the prevention and treatment of various diseases, and its therapeutic effects are documented extensively in the classical medical literature [34].

In this study, two HCHs (Scutellariae Radix and Coptidis Rhizoma) were investigated using network pharmacology to explore their therapeutic potential for CHHF, in comparison with two BTHs (Angelicae Sinensis Radix and Paeoniae Radix Alba), which are traditionally used to treat this condition. By integrating target protein data from TCMSP and GeneCards and visualizing the interaction networks using Venny and Cytoscape, potential target proteins associated with CHHF were identified for each herb.

Among the 81 proteins uniquely targeted by individual herbs, 61 were specific to Coptidis Rhizoma, while Angelicae Sinensis Radix had no unique targets. This difference may largely reflect disparities in the number of target proteins collected for each herb. Nevertheless, the fact that 18 of the 85 overlapping proteins were shared among Scutellariae Radix, Coptidis Rhizoma, and Paeoniae Radix Alba is significant, as it suggests that their common pharmacological effects are not solely determined by their classical categories (heat-clearing vs. blood-tonifying) but may also be influenced by their intrinsic properties and flavors (性味).

From the perspective of traditional herbal properties, Scutellariae Radix and Coptidis Rhizoma are both characterized as bitter and cold (苦寒), whereas the BTHs differ: Angelicae Sinensis Radix is warm and sweet–pungent (溫, 甘辛), while Paeoniae Radix Alba is slightly cold and bitter–sour (微寒, 苦酸), more similar to the HCHs [21]. This similarity in properties and flavors may partially explain the overlap in target protein distributions among these herbs.

At the pathway level, database-derived enrichment showed substantial overlap between Scutellariae Radix and Paeoniae Radix Alba. Targets associated with the HCHs also showed prominent representation in hemostasis- and coagulation-related pathways, including platelet activation, signaling and aggregation, integrin signaling, and fibrin clot formation. Because pathway enrichment reflects the statistical distribution of associated targets rather than direct pathway activity, these findings should be interpreted as candidate pathway associations rather than demonstrated pharmacological effects.

In the present Reactome analysis, targets associated with Scutellariae Radix, Coptidis Rhizoma, and Paeoniae Radix Alba were enriched in MAPK-related signaling pathways. This represents a pathway-enrichment finding based on database-derived target associations and does not indicate activation or inhibition of MAPK signaling by these herbs.

Previous studies have implicated p38 MAPK signaling in vascular responses to inflammatory cytokines and oxidative stress, including altered nitric oxide (NO) availability, endothelin-1 expression, reactive oxygen species (ROS) accumulation, and responses of VSMCs and ECs [12]. These mechanisms are biologically relevant to endothelial dysfunction and sustained peripheral vasoconstriction, features that are also relevant to CHHF and RP.

Taken together, the overlap between the predicted herb-associated targets and MAPK-related pathways raises the hypothesis that MAPK-associated vascular stress responses may contribute to the biological relevance of these herbs to CHHF. However, the present network pharmacology analysis cannot establish whether these herbs activate, inhibit, or otherwise modulate MAPK signaling, and this proposed relationship requires experimental validation.

In addition, Coptidis Rhizoma was found to be enriched in the signal transduction pathway, including targets associated with CXCL11 signaling. It is established that CXCR3–CXCL11 signaling contributes to vascular stabilization by suppressing excessive endothelial cell proliferation and promoting pericyte recruitment [13]. Given that microvascular instability and reduced peripheral blood flow are key pathological features of CHHF, this raises the hypothesis that Coptidis Rhizoma may exert vascular-stabilizing effects via CXCL11-associated signaling, warranting further experimental validation. Accordingly, not only vasodilation but also vascular structural stability and the suppression of aberrant EC proliferation may represent an important, previously underexplored therapeutic factor in CHHF management.

Moreover, in the pathophysiological mechanisms underlying dysautonomia-related CHHF, the role of α2-ARs has been highlighted as a key contributor [8]. It is established that cutaneous vasculature is more sensitive to α2-ARs than to α1-adrenergic receptors (α1-ARs), that the α2A-AR subtype mediates rapid cutaneous vasoconstriction in response to sympathetic stimulation, and that α2C-ARs, although normally quiescent, translocate to the cell membrane upon cold stimulation and rapidly amplify the contractile response [14,15,35]. In the present network pharmacology analysis, *ADRA2C* (encoding α2C-AR) was identified as a target of Coptidis Rhizoma, while *ADRA2A* (encoding α2A-AR) was identified as a shared target of Scutellariae Radix and Angelicae Sinensis Radix; Scutellariae Radix was additionally classified into the platelet activation, signaling, and aggregation pathway via ADRA2A. These findings raise the hypothesis that Coptidis Rhizoma and Scutellariae Radix are associated with α2-AR-mediated, cold-induced vasoconstrictive mechanisms—and, for Scutellariae Radix, concurrently with platelet activation—representing a candidate mechanism underlying the cold hypersensitivity and vasospasm observed in CHHF and RP.

The HCHs showed the highest proportions of pathways related to the immune system and signal transduction, including those involving nuclear factor kappa-light-chain-enhancer of activated B cells (NF-κB), Janus kinase–signal transducer and activator of transcription (JAK-STAT), interleukin-4 and interleukin-13 (IL-4/13), and IFN-γ signaling, suggesting potential relevance to anti-inflammatory and cytokine-modulating processes. Notably, the identification of inflammatory cytokine targets such as TNF-α, IFN-γ, interleukin-16 (IL-16), and members of the interleukin-1 (IL-1) family in the present study is consistent with previous reports indicating that angiopoietin-2 (Ang-2) destabilizes ECs in the pathophysiology of RP, thereby rendering them more susceptible to inflammatory cytokine stimulation and inducing endothelial cell apoptosis, which ultimately exacerbates microvascular damage and ischemia [10]. Conversely, the BTHs exhibited distinct patterns. Paeoniae Radix Alba showed the highest proportion of immune system-related pathways (38%), with enriched targets involved in TNF signaling, TIR-domain-containing adapter-including interferon-β-mediated cell death (TRIF-mediated cell death), and IκB kinase (IKK) recruitment, suggesting a potential association with TNF-mediated inflammation and apoptosis. In contrast, Angelicae Sinensis Radix showed the lowest proportion of immune pathways (14%) but the highest representation in metabolic (27%) and neuronal (18%) pathways, indicating a relatively higher representation of pathways related to energy metabolism and mitochondrial function, suggesting a potential association with blood flow regulation.

These results suggest that HCHs and BTHs exhibit distinct but partially overlapping molecular profiles associated with CHHF, with HCHs showing greater representation of inflammation-, cytokine-, and hemostasis-related pathways, whereas BTHs showed associations with metabolic, neurovascular, and TNF-related pathways.

The term 淸熱藥 (HCHs) derives from the Chinese characters “淸” (to clear) and “熱” (heat), literally meaning “to clear heat.” In traditional theory, “heat” refers not only to fever but also to pathological states resembling inflammation. Many HCHs have been reported to have anti-inflammatory effects via multi-target mechanisms, providing an important pharmacological rationale for their traditional indications [35].

Traditionally, CHHF in KM has been categorized as cold pattern (寒證), including upper body heat and lower body cold (上熱下寒) or systemic coldness. However, the present study aimed to reinterpret CHHF from a new perspective. Conditions such as RP are characterized by excessive vasoconstriction or circulatory impairment of peripheral vessels triggered by cold exposure or emotional stress, often followed by reactive hyperemia accompanied by pain or redness. This pattern suggests that CHHF cannot be fully explained by the cold pattern alone.

Given that vascular constriction and reperfusion can elicit inflammatory responses in patients with CHHF [3,36], the anti-inflammatory effects of HCHs may alleviate these pathological reactions, thereby contributing to their therapeutic efficacy.

This study has several limitations.

Firstly, the scope of herbal selection was limited to four representative herbs, and the PubMed-based selection of HCHs yielded a relatively limited dataset. This may have excluded other potentially relevant herbs, particularly given the scarcity of previous studies investigating HCHs for CHHF. Further studies encompassing a broader range of herbal categories are warranted.

Secondly, although Angelicae Gigantis Radix is commonly used in Korean clinical practice, data for this herb were unavailable in the TCMSP database. Therefore, Angelicae Sinensis Radix, which is traditionally used as a BTH in Chinese medicine, was included in the present network pharmacology analysis. However, as these two herbs are botanically and chemically distinct, the findings derived from Angelicae Sinensis Radix cannot be directly generalized to Angelicae Gigantis Radix used in Korean clinical practice. Accordingly, this substitution represents a limitation of the present study, and future studies using species-specific phytochemical and target data for Angelicae Gigantis Radix are warranted.

Thirdly, the compound–target and disease-associated gene datasets were derived from TCMSP and GeneCards, respectively. TCMSP integrates herbal ingredients, ADME-related properties, and compound–target information, while GeneCards consolidates gene-centered information from diverse biomedical sources; these features enable systematic data retrieval and an internally consistent analytical workflow [37,38]. However, TCMSP includes both experimentally validated and SysDT-predicted compound–target associations and may not fully capture recently reported or poorly characterized constituents [37,39,40]. Likewise, GeneCards relevance scores integrate heterogeneous, source-dependent evidence and may preferentially prioritize well-studied genes [38,41]. In addition, active compounds were selected using predefined thresholds of OB ≥ 30% and DL ≥ 0.18. Although these thresholds were adopted based on their widespread use in previous network pharmacology studies, thereby facilitating comparability with earlier research, different cutoff values could alter the number and composition of the selected compounds and exclude pharmacologically relevant constituents. These database- and threshold-dependent factors may subsequently influence the PPI and pathway-enrichment results. Future studies incorporating cross-database analyses, alternative screening criteria, and sensitivity analyses are warranted to assess the robustness of the findings.

Fourthly, the present study analyzed individual herbs rather than multi-herb formulas commonly used in clinical practice. Although this approach was selected to facilitate direct comparison between heat-clearing and blood-tonifying herb categories, it does not fully reflect the complexity of actual herbal prescriptions. Therefore, the findings should not be directly generalized to the therapeutic effects of multi-herb formulas.

Fifthly, this study was based solely on literature-derived network pharmacology analysis, including target prediction and pathway enrichment, without experimental or clinical validation. As pathway enrichment reflects the statistical distribution of predicted targets across biological pathways rather than a direct measurement of pathway activation or inhibition, the biological mechanisms proposed in this study should be interpreted as hypothesis-generating rather than confirmatory. Nonetheless, this analysis provides meaningful preliminary evidence supporting the potential relevance of HCHs to CHHF-related pathways, warranting further experimental and clinical investigation to validate the proposed mechanisms.

This study is the first to explore the therapeutic potential of HCHs (Scutellariae Radix and Coptidis Rhizoma) by comparing them with traditionally used BTHs (Angelicae Sinensis Radix and Paeoniae Radix Alba) for CHHF treatment. The principal novelty of this study lies in extending the conventional therapeutic perspective on CHHF beyond the herbal categories traditionally associated with its treatment: CHHF has generally been interpreted within a cold-related pathological framework in KM, and blood-tonifying or warming therapeutic approaches have consequently occupied a central role in its clinical management, whereas HCHs, traditionally characterized by their heat-clearing actions and predominantly cold nature, have received relatively little attention as potential therapeutic candidates for CHHF. By directly comparing representative HCHs with clinically relevant BTHs within the specific context of CHHF, the present study identified HCH-associated molecular pathways related to hemostasis, inflammatory signaling, and vascular regulation, together with both distinct and overlapping pathway profiles relative to BTHs, revealing novel mechanisms of Scutellariae Radix and Coptidis Rhizoma not explained by the classical actions of BTHs and providing new insights into the pharmacological basis of CHHF treatment. Therefore, the originality of this study lies in its pathway-based comparative approach to investigating the counterintuitive possibility that HCHs may also have molecular relevance to a condition conventionally approached through blood-tonifying and warming strategies. This category-level comparison broadens the pharmacological perspective on CHHF and provides a hypothesis-generating basis for further investigation of HCHs as potentially complementary therapeutic candidates.

Furthermore, by integrating compound–target interaction data from public databases and applying network pharmacology analysis, this study revalidated traditional empirical knowledge of herbal efficacy through modern bioinformatics approaches. This integration bridges the gap between traditional concepts and molecular-level mechanisms, highlighting the importance of combining empirical and mechanistic evidence in future KM research.

## 5. Conclusions

Network pharmacology analysis was conducted on two HCHs (Scutellariae Radix and Coptidis Rhizoma) and two BTHs (Angelicae Sinensis Radix and Paeoniae Radix Alba) commonly prescribed for CHHF in KM.

The results demonstrated that Scutellariae Radix and Paeoniae Radix Alba shared a substantial number of signaling pathways, while the former uniquely contained additional pathways related to hemostasis and coagulation.

Overall, the predicted targets of HCHs showed greater representation in inflammation-, cytokine-, and hemostasis-related pathways, whereas those of BTHs showed greater representation in pathways potentially associated with blood flow and microcirculation.

Although this study demonstrated the potential binding affinity between active compounds and predicted protein targets, a limitation of this work is that molecular docking and dynamics simulations were not performed to evaluate the dynamic stability of the ligand-receptor complexes. Moreover, this study has limitations regarding the interpretation of computational predictions, screening thresholds, and discussion of database limitations. 

These findings are hypothesis-generating and suggest that HCHs such as Scutellariae Radix and Coptidis Rhizoma may be associated with biological pathways relevant to CHHF; however, their therapeutic effects and clinical applicability require further wet-lab experimental and clinical validation.

## Figures and Tables

**Table 1:** Centrality measures used for the selection of core genes in herbal medicine.

	BC	CC	DC	EC
Scutellariae Radix	12.0	0.216106	7.724	0.062662
Coptidis Rhizoma	47.0	0.178791	9.786	0.04442
Angelicae Sinensis Radix	1.5	0.191165	2.825	0.057668
Paeoniae Radix Alba	10.0	0.132553	6.13	0.074147

**Table 2:** Frequency of PubMed hits for candidate HCHs.

No.	Herbal Medicine Name AND (Keyword)	PubMed Frequency
1	Scutellariae Radix	27
2	Coptidis Rhizoma	10
3	Rehmanniae Radix	6
4	Gardeniae Fructus	2

**Table 3:** Active compounds and predicted protein targets of selected herbs from TCMSP.

Herbal Medicine	Number of Active Compounds	Number of Predicted Protein Targets (with Duplication)	Number of Predicted Protein Targets (Duplicates Removed)
Scutellariae Radix	35	507	124
Coptidis Rhizoma	11	284	182
Angelicae Sinensis Radix	2	69	53
Paeoniae Radix Alba	13	123	92

**Table 4:** UniProt search results and final determination of IDs for proteins with ambiguous matches.

Target Name	Search Results	Final Determination
Bcl-2-binding component 3	Isoforms 1/2 and 3/4 were retrieved	Isoform 1/2 was determined as correct
Calmodulin	CALM1, CALM2, CALM3 were retrieved, corresponding to three UniProt IDs: ‘P0DP23’, ‘P0DP24’, ‘P0DP26’	It was determined that Calmodulin corresponds to a total of three UniProt IDs
Coagulation Factor Xa	Retrieved as Coagulation Factor X	Determined as the UniProt ID of Coagulation Factor X
Thrombin	Retrieved as Prothrombin	Determined as the UniProt ID of Prothrombin

**Table 5:** Number of herb-derived targets and overlapping targets with CHHF.

Herbal Medicine	Number of Herb-Derived Target Proteins	Number of Overlapping Target Proteins with CHHF
Scutellariae Radix	124	84
Coptidis Rhizoma	289	138
Angelicae Sinensis Radix	53	35
Paeoniae Radix Alba	92	66

**Table 6:** Number of nodes and core genes of selected herbs based on centrality measures.

Herbal Medicine	Number of Overlapping Protein Targets with CHHF	PPI Network Analysis			Core Gene Analysis	
		Number of Nodes	Number of Edges	Average Node Degree	Number of Nodes	Number of Core Genes
Scutellariae Radix	84	83	438	10.6	77	24
Coptidis Rhizoma	138	137	1132	16.5	134	41
Angelicae Sinensis Radix	35	35	56	3.2	28	9
Paeoniae Radix Alba	66	66	232	7.03	57	17

**Table 7:** Pathway enrichment results of common targets between CHHF and selected herbs based on Reactome analysis.

Pathway Category	Scutellariae Radix	Coptidis Rhizoma	Angelicae Sinensis Radix	Paeoniae Radix Alba	Pathways Enriched by ≥1 Herb
Hemostasis	9	9	0	4	13
Immune System	40	62	6	55	68
Metabolism	33	27	12	29	38
Neuronal System	10	9	8	6	14
Signal Transduction	61	81	18	50	100
Total Pathways	153	188	44	144	

## Data Availability

The original contributions presented in this study are included in the article. Further inquiries can be directed to the corresponding authors. The raw data were derived from the following resources available in the public domain: TCMSP ((https://www.tcmsp-e.com/ (accessed on 2 August 2025)), GeneCards (https://www.genecards.org/ (accessed on 2 August 2025)), UniProt (https://www.uniprot.org/ (accessed on 2 August 2025)), STRING (https://string-db.org/ (accessed on 27 August 2025)), and Reactome (https://reactome.org/ (version 93; accessed on 25 August 2025).

## Abbreviations

The following abbreviations are used in this manuscript:

RP	Raynaud’s phenomenon
KM	Korean medicine
CHHF	Cold hypersensitivity in hands and feet
TCM	Traditional Chinese medicine
HCHs	Heat clearing herbs
ECs	Endothelial cells
TNF-α	Tumor necrosis factor-α
IFN-γ	Interferon-γ
P38 MAPK	P38 mitogen-activated protein kinase
VSMCs	Vascular smooth muscle cells
NO	Nitric oxide
ROS	Reactive oxygen species
α2-ARs	α2-adrenergic receptors
α1-ARs	α1-adrenergic receptors
α2A-AR	α2A-adrenergic receptors
α2C-AR	α2C-adrenergic receptors
NCKM	National Clinical Practice Guidelines for Korean Medicine
TCMSP	Traditional Chinese Medicine Systems Pharmacology Database and Analysis Platform
PPI	Protein–protein interaction
STRING	Search tool for the retrieval of interacting genes/proteins
CytoNCA	Cytoscape network centrality analysis
CPG of CHHF	Clinical practice guideline of Korean medicine for cold hypersensitivity in hands and feet
ADME	Absorption, distribution, metabolism, excretion
BC	Betweenness centrality
CC	Closeness centrality
DC	Degree centrality
EC	Eigenvector centrality
CPG	Clinical practice guideline
BTHs	Blood tonifying herbs
FDR	False discovery rate
SR	Scutellariae Radix
CR	Coptidis Rhizoma
ASR	Angelicae Sinensis Radix
PRA	Paeoniae Radix Alba
ADRA2C	Adrenoceptor Alpha 2C
NF-κB	Nuclear factor kappa-light-chain-enhancer of activated B cells
JAK-STAT	Janus kinase–signal transducer and activator of transcription
IL-4/13	Interleukin-4 and Interleukin-13
IL-16	Interleukin-16
IL-1	Interleukin-1
TRIF-mediated cell death	TIR-domain-containing adapter-inducing interferon-β-mediated cell death
IKK	IκB kinase

## Appendix A

### Appendix A.1

The active compounds listed in Table A1, Table A2, Table A3 and Table A4 were retrieved from the TCMSP ((https://www.tcmsp-e.com/ (accessed on 2 August 2025)) and the PubChem database (https://pubchem.ncbi.nlm.nih.gov (accessed on 2 August 2025)). For compounds not available in TCMSP or requiring structural confirmation, chemical information including PubChem CID, molecular formula, molecular weight, and canonical structure was obtained from PubChem. Structural data were cross-validated to ensure consistency between databases. Duplicated entries were removed, and compounds lacking definitive structural or molecular information were excluded. Their biological activities and key findings from the PubMed database (https://pubmed.ncbi.nlm.nih.gov (accessed on 2 August 2025)) are reported in Table A5.

**Table A1:** Active compounds of Scutellariae Radix.

No.	Molecule Name	PubChem CID	Molecular Formula	Structure	Molecular Weight (g/mol)
1	(2R)-7-hydroxy-5-methoxy-2-phenylchroman-4-one	821279	C_16_H_14_O_4_		270.28 g/mol
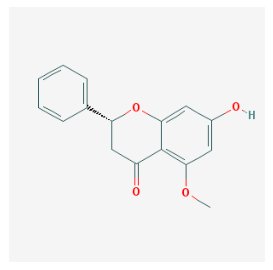
2	11,13-Eicosadienoic acid, methyl ester	5365674	C_21_H_38_O_2_		322.5 g/mol
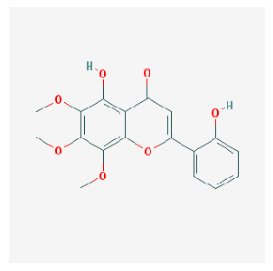
3	5,2′-Dihydroxy-6,7,8-trimethoxyflavone	159029	C_18_H_16_O_7_		433.3 g/mol
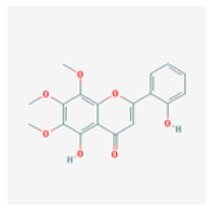
4	5,2′,6′-Trihydroxy-7,8-dimethoxyflavone	5322059	C_23_H_22_O_13_		506.4 g/mol
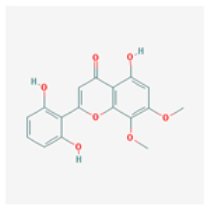
5	5,7,2,5-tetrahydroxy-8,6-dimethoxyflavone	44258627	C_17_H_18_O_8_		350.3 g/mol
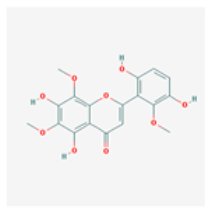
6	5,7,2′,6′-Tetrahydroxyflavone	5321865	C_21_H_20_O_11_		448.4 g/mol
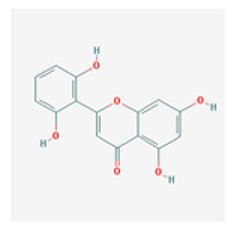
7	5,7,4′-trihydroxy-6-methoxyflavanone	5322074	C_16_H_14_O_6_		302.28 g/mol
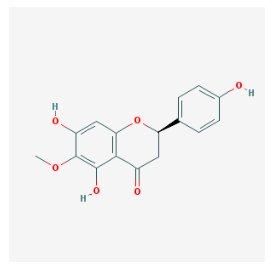
8	5,7,4′-trihydroxy-8-methoxyflavanone	26213330	C_16_H_14_O_6_		302.28 g/mol
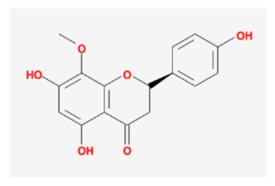
9	5,7,4′-Trihydroxy-8-methoxyflavone	5322078	C_16_H_12_O_6_		300.26 g/mol
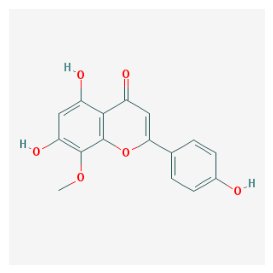
10	5,8,2′-Trihydroxy-7-methoxyflavone	156992	C_16_H_12_O_5_		300.26 g/mol
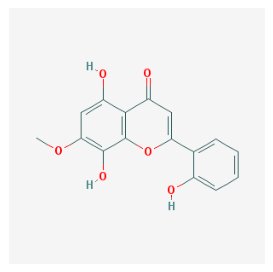
11	Acacetin	5280442	C_16_H_12_O_5_		284.26 g/mol
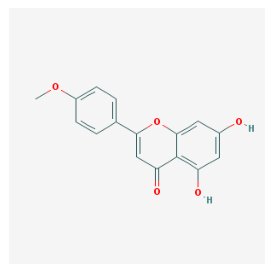
12	Baicalein	5281605	C_15_H_10_O_5_		270.24 g/mol
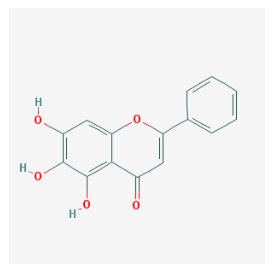
13	Beta-sitosterol	222284	C_29_H_50_O		414.7 g/mol
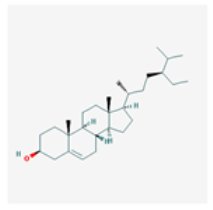
14	bis[(2S)-2-ethylhexyl] benzene-1,2-dicarboxylate	7057920	C_24_H_38_O_4_		390.6 g/mol
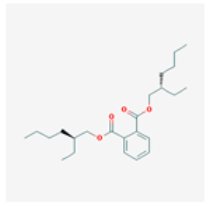
15	Carthamidin	188308	C_15_H_12_O_6_		288.25 g/mol
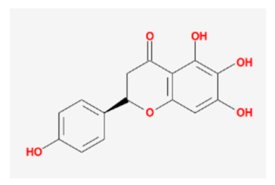
16	Coptisine	72322	C_19_H_14_NO_4_^+^		320.3 g/mol
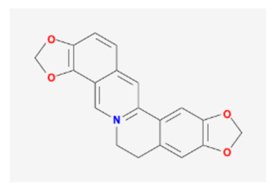
17	Dihydrobaicalin_qt	14135323	C_15_H_12_O_5_		272.25 g/mol
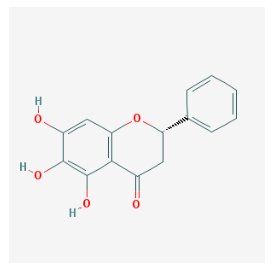
18	Dihydrooroxylin	25721350	C_16_H_14_O_5_		286.28 g/mol
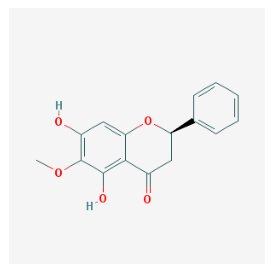
19	Dihydrooroxylin A	177032	C_16_H_14_O_5_		286.28 g/mol
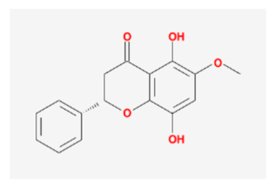
20	Diop (diisooctyl phthalate)	33934	C_24_H_38_O_4_		390.6 g/mol
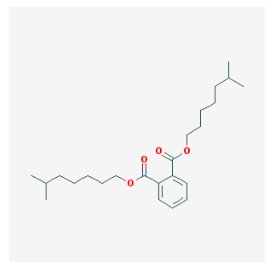
21	ent-Epicatechin	182232	C_15_H_14_O_6_		290.27 g/mol
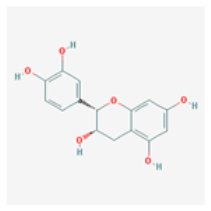
22	Epiberberine	160876	C_20_H_18_NO_4_^+^		336.4 g/mol
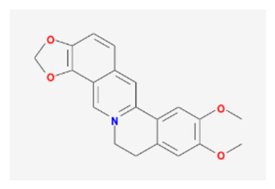
23	Eriodyctiol (flavanone)	373261	C_15_H_12_O_6_		288.25 g/mol
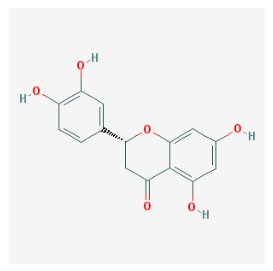
24	Moslosooflavone	188316	C_17_H_14_O_5_		298.29 g/mol
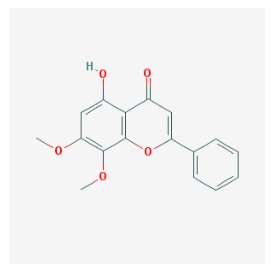
25	Neobaicalein	124211	C_19_H_18_O_8_		374.3 g/mol
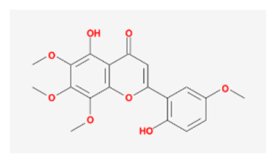
26	Norwogonin	5281674	C_15_H_10_O_5_		270.24 g/mol
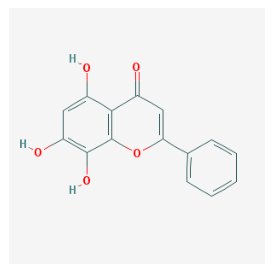
27	Oroxylin a	5320315	C_16_H_12_O_5_		284.26 g/mol
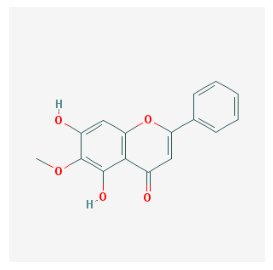
28	Panicolin	5320399	C_17_H_14_O_6_		314.29 g/mol
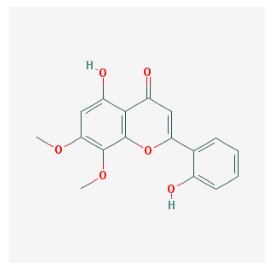
29	Rivularin	13889022	C_18_H_16_O_7_		344.3 g/mol
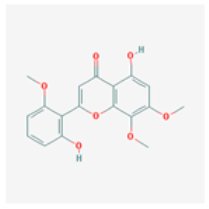
30	Salvigenin	161271	C_18_H_16_O_6_		328.3 g/mol
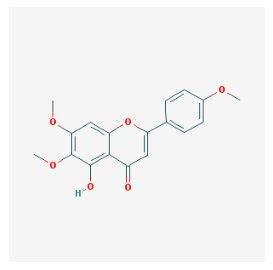
31	Sitosterol	12303645	C_29_H_50_O		414.7 g/mol
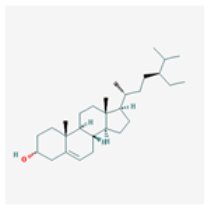
32	Skullcapflavone II	124211	C_19_H_18_O_8_		374.3 g/mol
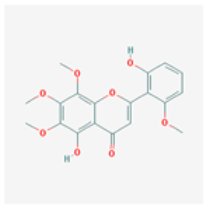
33	Stigmasterol	5280794	C_29_H_48_O		412.7 g/mol
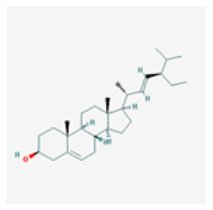
34	Supraene	638072	C_30_H_50_		410.7 g/mol
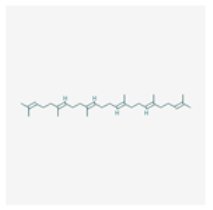
35	Wogonin	5281703	C_16_H_12_O_5_		284.26 g/mol
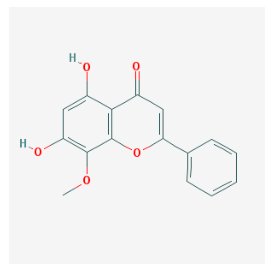

**Table A2:** Active compounds of Coptidis Rhizoma.

No.	Molecule Name	PubChem CID	Molecular Formula	Structure	Molecular Weight (g/mol)
1	(R)-Canadine	443422	C_20_H_21_NO_4_		339.4 g/mol
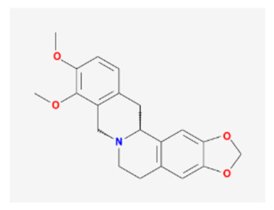
2	Berberrubine	72704	C_19_H_16_NO_4_^+^		322.3 g/mol
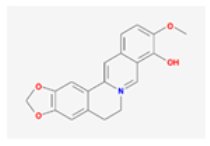
3	Berlambine	11066	C_20_H_17_NO_5_		351.4 g/mol
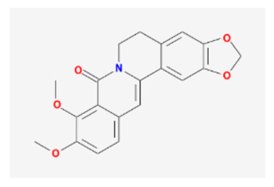
4	Coptisine	72322	C_19_H_14_NO_4_^+^		320.3 g/mol
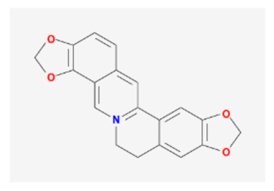
5	Epiberberine	160876	C_20_H_18_NO_4_^+^		336.4 g/mol
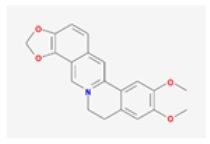
6	Magnograndiolide	5319198	C_15_H_22_O_4_		266.33 g/mol
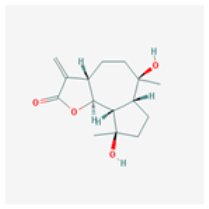
7	Moupinamide	5280537	C_18_H_19_NO_4_		313.3 g/mol
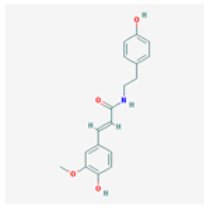
8	Obacunone	119041	C_26_H_30_O_7_		454.5 g/mol
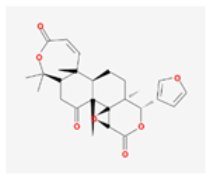
9	Palmatine	19009	C_21_H_22_NO_4_^+^		352.4 g/mol
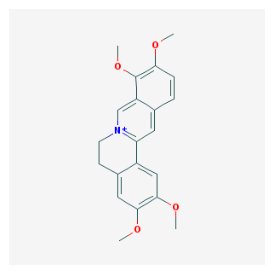
10	Quercetin	5280343	C_15_H_10_O_7_		302.23 g/mol
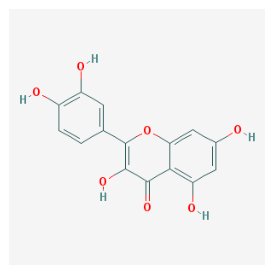
11	Worenine	20055073	C_20_H_16_NO_4_^+^		334.3 g/mol
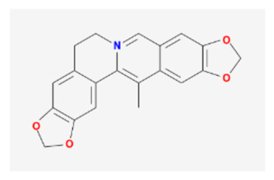

**Table A3:** Active compounds of Angelicae Sinensis Radix.

No.	Molecule Name	PubChem CID	Molecular Formula	Structure	Molecular Weight (g/mol)
1	Beta-sitosterol	222284	C_29_H_50_O		414.7 g/mol
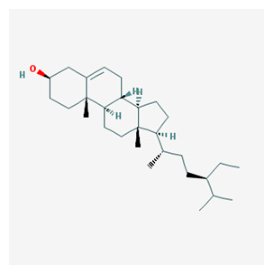
2	Stigmasterol	5280794	C_29_H_48_O		412.7 g/mol
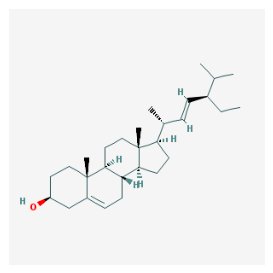

**Table A4:** Active compounds of Paeoniae Radix Alba.

No.	Molecule Name	PubChem CID	Molecular Formula	Structure	Molecular Weight (g/mol)
1	(+)-catechin	9064	C_15_H_14_O_6_		290.27 g/mol
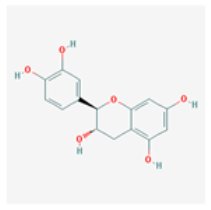
2	(3S,5R,8R,9R,10S,14S)-3,17-dihydroxy-4,4,8,10,14-pentamethyl-2,3,5,6,7,9-hexahydro-1H-cyclopenta[a]phenanthrene-15,16-dione	9841735	C_22_H_30_O_4_		358.5 g/mol
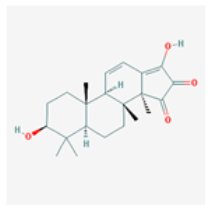
3	11alpha,12alpha-epoxy-3beta-23-dihydroxy-30-norolean-20-en-28,12beta-olide		C_29_H_42_O_5_		470.71 g/mol
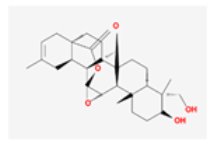
4	Albiflorin_qt		C_17_H_18_O_6_		318.35 g/mol
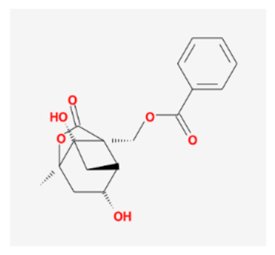
5	Benzoyl paeoniflorin		C_30_H_32_O_12_		584.62 g/mol
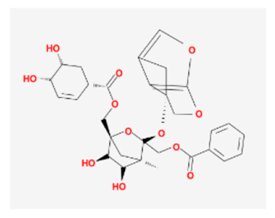
6	Beta-sitosterol	222284	C_29_H_50_O		414.7 g/mol
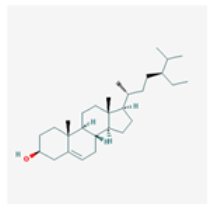
7	Kaempferol	5280863	C_15_H_10_O_6_		286.24 g/mol
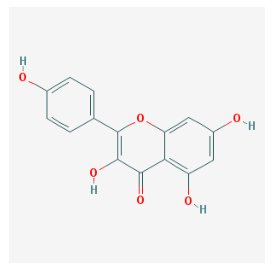
8	Lactiflorin	5318917	C_23_H_26_O_10_		462.4 g/mol
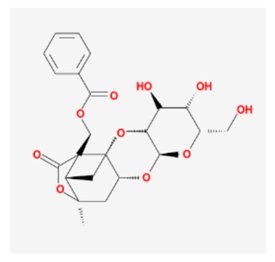
9	Mairin (betulinic acid)	64971	C_30_H_48_O_3_		456.7 g/mol
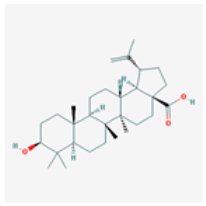
10	Paeoniflorigenone	133475	C_17_H_18_O_6_		318.35 g/mol
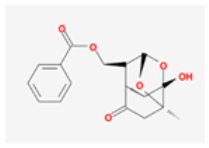
11	Paeoniflorin	442534	C_23_H_28_O_11_		480.51 g/mol
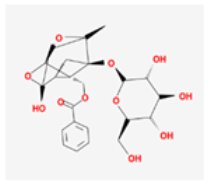
12	Paeoniflorin_qt		C_17_H_18_O_6_		318.35 g/mol
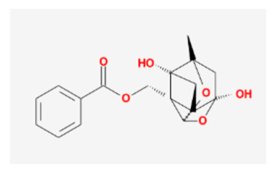
13	3-epi-beta-sitosterol	12303645	C_29_H_50_O		414.7 g/mol
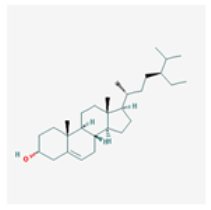

**Table A5:** The important pharmacological activities and key findings of selected active compounds related to CHHF.

Active Compounds	Biological Activity	Key Findings & Results	References
5,2′-Dihydroxy-6,7,8-trimethoxyflavone	Anti-inflammatory	Inhibited STAT3 expression by downregulating the PIK3R1/SRC pathway in Neuro 2A cells.	[42]
5,7,2,5-tetrahydroxy-8,6-dimethoxyflavone	Anti-inflammatory (neuroinflammation)	Promoted miR-145 expression and down-regulation of PI3K/AKT signaling pathway in MKN28 cells.	[43]
Acacetin	Blood flow (cardiovascular) Anticoagulant Anti-inflammatory	Suppressing oxidative stress, reducing inflammation, and preventing cardiomyocyte apoptosis and endothelial cell injury. Prolonging prothrombin time, thromboplastin time, and thrombin time and reducing fibrinogen content. Suppressed the expression of lipopolysaccharide (LPS)-activated phosphorylated ERK (p-ERK), p-JNK, and p-p38, inhibited NF-κB p65 phosphorylation and nuclear translocation.	[44,45,46]
Baicalein	Anti-inflammatory Blood flow Hemostasis	Attenuated serum levels of cytokines (interleukin (IL)-1β, IL-2, IL-4, IL-6, IL-17A, tumor necrosis factor-α), chemokines (monocyte chemoattractant protein-1, macrophage inflammatory protein-1 beta) Blocked TNF-α-induced NF-κB activation. Improved heart cell survival and overall cardiac function. Reduced the bleeding time, clotting time, and uterine bleeding.	[47,48,49,50]
Beta-sitosterol	Anti-inflammatory (blood flow)	Attenuating lipid accumulation, inflammation, and oxidative stress via coordinated regulation of the MAPK/Nrf2/NLRP3 pathways. Activating the hypoxia-inducible factor-1(HIF-1_)/mammalian target of rapamycin(mTOR) signaling pathway	[51,52]
Coptisine	Anti-inflammatory	Regulating the signaling transduction of pathways such as NF-κB, MAPK, PI3K/Akt, NLRP3 inflammasome. Restraining reactive oxygen species (ROS), malondialdehyde (MDA), tumor necrosis factor (TNF)-α, interleukin (IL)-1β, IL-18 levels, down-regulating protein expressions of cleaved-caspase 3, apoptosis-inducing factor (AIF).	[53,54]
Eriodyctiol	Anticoagulant	Inhibited thrombin-induced platelet activation and decreased formation of reactive oxygen species in activated platelets.	[55]
Norwogonin	Anti-inflammatory	Inhibited the Src/AKT1/NF-κB signaling pathway.	[56]
Oroxylin A	Anticoagulant	Suppressed microvascular thrombosis and platelet infiltration.	[57]
Stigmasterol	Anti-inflammatory	Inhibited the TGF-β1/Smad2 and IL-17A signaling pathways.	[58]
Wogonin	Anti-inflammatory Anticoagulant	Blockade of NLRP3/Caspase-1/GSDMD pathway. Inhibited ERK/Egr-1- and JNK/AP-1-mediated transactivation of TF promoter activity.	[59,60]
Quercetin	Anti-inflammatory Anticoagulant	Modulating NOX2/ROS/NF-kB in lung epithelial cells Attenuated atherosclerosis via modulating oxidized LDL.	[61,62]
(+)-catechin	Anti-inflammatory (blood flow)	Attenuated atherosclerotic plaque burden and inflammation. Attenuated inflammatory response triggered by TNF-α through signaling cascades involved in inflammation.	[63,64]
Kaempferol	Anti-inflammatory	Shifted polarization from pro-inflammatory M1 to anti-inflammatory M2 phenotypes. Attenuates atherosclerosis via the PI3K/AKT/Nrf2 pathway.	[65,66]
Paeoniflorin	Anti-inflammatory Anticoagulant	Inhibited PI3K/Akt/ERK-mediated HIF-1α and TLR4/MyD88/NF-κB inflammatory signaling. Inhibited the expression of TNF-α by suppressing the NF-κB signaling pathway.	[67,68]

**Table A6:** Overlapping target proteins between CHHF and four selected herbs visualized by Cytoscape.

Set of Target Proteins	Name of Target Proteins
Targets unique to Scutellariae Radix	PRKCD FASLG FN1 CYP19A1 KDR MAPK14 CYCS CYP2C9 CA2 FASN MCL1 PTPN1 FABP5 CDK7 FOSL1
Targets unique to Coptidis Rhizoma	IL10 PTEN IFNG EGFR IL1B SOD1 CD40LG CRP GJA1 IL2 COL1A1 ERBB2 MAPK1 NFKBIA MYC MMP2 HSPB1 RAF1 EGF THBD CTSD COL3A1 IL1A CAV1 CHEK2 RASA1 NCF1 RUNX2 SERPINE1 PARP1 SPP1 NFE2L2 ERBB3 CXCL10 CHUK PLAT POR GRP78 BCL2L1 IGFBP3 MMP3 IRF1 F3 PPARA BIRC5 GRIA2 TOP1 ODC1 ABCG2 E2F1 NQO1 HTR3A RASSF1 MGAM PRKCB HSF1 OPRD1 CXCL11 HK2 ADRA2C ADRA1D
Targets unique to Paeoniae Radix Alba	IKBKB CAT MAPK8 CD14 SLPI
Common targets of Scutellariae Radix and Coptidis Rhizoma	TP53 IL8 VEGFA MPO CCL2 MMP9 IGF2 CCND1 HIF1A CDKN1A FOS ESR2 CHEK1 CDK2 CCNB1 PPARD
Common targets of Scutellariae Radix and Angelicae Sinensis Radix	MAOA ADRB1 ADRA2A NCOA1 CTRB1
Common targets of Coptidis Rhizoma and Paeoniae Radix Alba	STAT1 ICAM1 INSR HMOX1 CYP3A4 GSTM1 XDH VCAM1 CYP1B1 GSTP1 SELE CYP1A1 SLC2A4 ALOX5AP NR1I2 NR1I3
Common targets of Scutellariae Radix and Coptidis Rhizoma and Angelicae Sinensis Radix	SLC6A3 MAOB AKR1B1
Common targets of Scutellariae Radix and Coptidis Rhizoma and Paeoniae Radix Alba	TNF IL6 AKT1 ESR1 PPARG AR F2 NOS3 MMP1 NOS2 ACHE CALM1 RELA CYP1A2 CDC2 AHR TOP2A DPP4
Common targets of Scutellariae Radix and Angelicae Sinensis Radix and Paeoniae Radix Alba	NR3C2 PGR CHRNA7
Common targets of Scutellariae Radix and Coptidis Rhizoma and Angelicae Sinensis Radix and Paeoniae Radix Alba	TGFB1 CASP8 SCN5A PTGS2 BCL2 PIK3CG CASP3 JUN BAX SLC6A4 PRKACA ADRB2 PON1 HTR2A PRKCA CASP9 OPRM1 GABRA1 PTGS1 HSP90AB1 CHRM3 DRD1 ADRA1A ADRA1B

**Table A7:** Overlapping core genes between CHHF and four selected herbs visualized by Cytoscape.

Set of Core Genes	Name of Core Genes
Targets unique to Scutellariae Radix	FN1
Targets unique to Coptidis Rhizoma	PRKCA MYC EGFR MAPK1 ERBB2 PTEN NFE2L2 SPP1 CAV1 RAF1 IL1B RUNX2 TGFB1 VCAM1 NFKBIA BCL2L1 PARP1 MMP3
Targets unique to Angelicae Sinensis Radix	CASP9
Targets unique to Paeoniae Radix Alba	MAPK8 CALM3 NOS3
Common targets of Scutellariae Radix and Coptidis Rhizoma	TP53 CXCL8 FOS MMP9 HIF1A CCL2 CDKN1A CCND1 CDK1
Common targets of Scutellariae Radix and Angelicae Sinensis Radix	PGR NCOA1
Common targets of Coptidis Rhizoma and Paeoniae Radix Alba	HMOX1 STAT1
Common targets of Scutellariae Radix and Coptidis Rhizoma and Angelicae Sinensis Radix	PTGS2 JUN HSP90AB1 PRKACA CASP3 BCL2
Common targets of Scutellariae Radix and Coptidis Rhizoma and Paeoniae Radix Alba	TNF PPARG ESR1 RELA AKT1 IL6

### Appendix A.2

Overlapping and unique target proteins between CHHF-related targets and the four selected herbs were identified using Venny 2.1.0. Targets were classified into herb-specific and shared target groups and visualized using Cytoscape.

### Appendix A.3

Overlapping and unique core genes between CHHF-related targets and the four selected herbs were identified using Venny 2.1.0. Targets were classified into herb-specific and shared target groups and visualized using Cytoscape.
